# Association Between Epinephrine Administration Timing and Outcomes in Adult Out-of-Hospital Cardiac Arrest: A Systematic Review and Dose–Response Meta-Analysis

**DOI:** 10.3390/medicina62081495

**Published:** 2026-08-03

**Authors:** Chiwon Ahn, Jae Hwan Kim, So June Hwang, Young Taeck Oh

**Affiliations:** 1 Department of Emergency Medicine, College of Medicine, Chung-Ang University, Seoul 06973, Republic of Korea; cahn@cau.ac.kr (C.A.); sult@cau.ac.kr (J.H.K.); 2 Department of Emergency Medicine, Chung-Ang University Hospital, Seoul 06973, Republic of Korea; junee3278@cauhs.or.kr

**Keywords:** out-of-hospital cardiac arrest, epinephrine, cardiopulmonary resuscitation, time-to-treatment, meta-analysis, systematic review

## Abstract

*Background and Objectives*: The optimal timing of epinephrine in out-of-hospital cardiac arrest (OHCA), and whether it differs by initial rhythm, is uncertain and rests almost entirely on confounded observational data. We synthesized this evidence, noting each study’s time origin. *Materials and Methods*: PubMed, Embase, and the Cochrane Central Register of Controlled Trials were searched from inception to 15 March 2026 for studies relating epinephrine timing to outcome in adult non-traumatic OHCA. Per-minute, categorical and dose–response random-effects meta-analyses were performed by rhythm, with ROBINS-I and GRADE appraisal. *Results*: Twenty-five studies were included. For non-shockable rhythm, each 1 min delay was associated with 4.2% lower survival odds (OR 0.958, 95% CI 0.949–0.968; k = 4, I^2^ = 0%), unchanged when restricted to studies sharing one time origin. Early epinephrine was associated with favorable neurological outcome (k = 9, OR 2.76, 95% CI 2.06–3.71; I^2^ = 88%), though the prediction interval crossed the null (0.98–7.79), and the shockable-versus-non-shockable difference did not persist under a common time origin (*p* = 0.46). Several estimates were biologically implausible (adjusted OR 5.92), indicating substantial residual confounding; GRADE certainty was very low. *Conclusions*: The per-minute survival gradient in non-shockable rhythm was the most internally consistent estimate—internally consistent rather than reliable, since studies sharing one structural bias can agree without being valid. Every estimate is almost certainly inflated by resuscitation time bias. The evidence supports minimizing avoidable delay but cannot define a timing threshold; trials randomizing timing and individual patient data meta-analyses with harmonized time definitions are needed.

## 1. Introduction

Out-of-hospital cardiac arrest (OHCA) remains a leading cause of death worldwide, with survival rates typically ranging from 5% to 15% [1]. Epinephrine has been a mainstay of cardiac arrest management, recommended for its ability to increase coronary perfusion pressure and facilitate return of spontaneous circulation (ROSC) [2,3]. The PARAMEDIC2 trial established that epinephrine improves 30-day survival compared with placebo, though at the cost of more survivors with severe neurological disability [4].

While whether to give epinephrine has been addressed by randomized evidence, when to give it remains informed largely by observational data. Current American Heart Association (AHA) and European Resuscitation Council (ERC) guidelines recommend epinephrine as soon as possible for non-shockable rhythms and after initial defibrillation attempts for shockable rhythms [2,3], with recent 2025 updates reaffirming early administration for non-shockable rhythms [5,6]. A network meta-analysis by Fernando et al. (2023) [7] demonstrated differential effects of epinephrine by rhythm subgroup but did not examine timing. The three-phase model of cardiac arrest [8] gives a specific reason to expect that timing does not act identically across rhythms. A patient found in a shockable rhythm is typically still in the electrical phase, in which defibrillation rather than pharmacotherapy is the outcome-determining intervention, so a vasopressor would be expected to matter mainly once that patient has passed into the circulatory phase; on this reasoning any timing effect for shockable rhythms is conditional on, and secondary to, prompt defibrillation. A non-shockable presentation, by contrast, usually indicates that the circulatory or metabolic phase has already been reached, where restoring coronary perfusion pressure is the principal therapeutic target and epinephrine is the intervention aimed most directly at it, predicting a steeper and less conditional timing gradient. Whether the timing–outcome relationship does in fact differ by initial rhythm has not been comprehensively synthesized. Several studies report mixed results: some find consistent benefit of earlier administration across all rhythms [9,10], while others suggest a benefit limited to non-shockable rhythms [11,12] or non-linear relationships [13,14].

The most recent synthesis, a 2020 meta-analysis by Ran et al. [15], pooled early-versus-late comparisons across mixed populations. The present review differs from it in six specific respects: (i) a from-inception search of three databases extended to March 2026, which adds cohorts published after that review, including Fukuda 2021 [9], Enzan 2022 [16], Kawakami 2024 [14] and five cohorts first reported in 2025 [17,18,19,20,21]; (ii) pooling stratified by initial rhythm; (iii) per-minute (continuous) estimates rather than dichotomized comparisons alone; (iv) two-stage dose–response and restricted cubic spline modeling of the time–response shape; (v) ROBINS-I risk-of-bias and GRADE certainty assessment; and (vi) explicit management of overlap among the Japanese nationwide registry cohorts, which are counted only once in any single pooled estimate. Yet all of this evidence derives from observational studies, in which patients who receive epinephrine later are, by virtue of not yet having achieved ROSC, systematically different from those treated earlier, a structural problem termed resuscitation time bias [22] that cannot be removed by meta-analytic pooling. We therefore conducted a comprehensive systematic review searching three databases from inception, aiming to: (1) quantify the per-minute timing–outcome association in adult OHCA; (2) determine whether this association differs between shockable and non-shockable rhythms; (3) characterize the time–response curve shape using pooled restricted cubic spline modeling; and (4) explicitly appraise the extent to which the observed associations reflect confounding rather than a causal timing effect.

## 2. Materials and Methods

### 2.1. Study Design and Registration

This systematic review was conducted and reported in accordance with the Preferred Reporting Items for Systematic Reviews and Meta-Analyses (PRISMA) 2020 guidelines (Table S1) [23]. The review was not registered, and no protocol was published or registered in advance; the boundary between prespecified and post hoc analysis therefore rests on our own account and cannot be verified externally, and we state it explicitly here. The review question, the eligibility criteria, the primary outcome (favorable neurological outcome), the secondary outcomes (survival to hospital discharge or 30 days and ROSC), and the intention to stratify all analyses by initial rhythm were fixed before data extraction. The dose–response and restricted cubic spline analyses were planned before extraction as exploratory secondary analyses, but their implementation details—knot placement, the restriction of the primary categorical analysis to direct adjusted comparisons, the registry-overlap rules, and the E-value and time-origin sensitivity analyses—were specified after the extracted data had been inspected and are post hoc. Every outcome and analysis undertaken is reported, including those that were null or that weakened the principal findings; no analysis was performed and omitted. The eligibility criteria and analytical methods are reported in full in this article and the Supplementary Materials.

### 2.2. Eligibility Criteria

We included studies of adult (≥18 years) non-traumatic OHCA patients treated by emergency medical services (EMS) that reported an association between timing of first epinephrine administration and at least one clinical outcome. The primary outcome was favorable neurological outcome (Cerebral Performance Category (CPC) 1–2 or modified Rankin Scale (mRS) ≤ 3; these thresholds are considered broadly equivalent for defining functional independence [24]). Secondary outcomes were survival to hospital discharge (or 30-day survival) and ROSC. Observational studies and secondary analyses of randomized trials were eligible. Studies comparing routes of administration or dosing intervals without time-to-first-dose analysis were excluded, as were case reports and case series (N < 100). Conference abstracts without peer-reviewed full text were excluded from quantitative synthesis but could be included in narrative synthesis if they met all other eligibility criteria.

### 2.3. Search Strategy

PubMed, Embase, and the Cochrane Central Register of Controlled Trials (CENTRAL) were searched from database inception through 15 March 2026, with no language or publication-date restriction. The search combined four concept blocks (cardiac arrest, epinephrine/vasopressors, timing/time-to-treatment, and outcomes) using both controlled vocabulary and free-text terms (complete strategies in Table S2). Pediatric, in-hospital, and animal records were removed at the screening stage rather than through search-level exclusion filters, after we determined that title/abstract exclusion terms (e.g., “NOT child”) risked discarding eligible mixed adult-and-pediatric cohorts. Hansen 2018 [10], for example, reports a combined pediatric-and-adult non-shockable cohort from which the adult stratum is separately extractable; a query-level “NOT child” filter would have removed that report from the search output altogether rather than flagging it for inspection. Screening at the record level rather than at the query level preserved such reports and kept every exclusion visible and auditable in the PRISMA flow diagram. Reference lists of all included studies and relevant systematic reviews, including the prior review by Ran et al. [15], were screened to identify additional eligible studies.

### 2.4. Study Selection and Data Extraction

Two reviewers (C.A. and J.H.K.) independently screened titles and abstracts and then full texts; disagreements were resolved by discussion, with a third reviewer (S.J.H.) adjudicating any that remained. Data were extracted by one reviewer (C.A.) and independently verified against the original publication by a second (J.H.K.); discrepancies were resolved by consensus. For studies reporting late-versus-early effects, odds ratios were inverted to a consistent early-versus-late direction. Risk of bias was assessed using the ROBINS-I tool (Risk Of Bias In Non-randomized Studies—of Interventions) [25], evaluating seven domains: confounding, selection of participants, classification of interventions, deviations from intended interventions, missing data, measurement of outcomes, and selection of reported results. Each domain was rated as low, moderate, serious, or critical risk of bias, with the overall judgment determined by the highest-risk domain.

### 2.5. Statistical Analysis

**Per-minute meta-analysis.** Studies reporting per-minute change in odds were pooled using inverse-variance random-effects meta-analysis (restricted maximum likelihood (REML)) on the log-OR scale.

**Categorical meta-analysis.** Studies comparing early versus late epinephrine were pooled using random-effects meta-analysis. There is no consensus threshold separating “early” from “late” epinephrine; we therefore used each study’s own dichotomization (or its most extreme timing categories) rather than imposing a uniform cut-point. These study-defined thresholds varied (ranging from approximately 10 to 20 min from the study’s time origin; e.g., a 19 min call-to-epinephrine cut-point in Tanaka 2016 [26] and ≤10 versus ≥20 min from CPR start in Funada 2018 [12]) and are detailed per study in Table 1 and Table S3. The primary analysis included only direct comparisons using adjusted effect estimates (adjusted ORs or propensity-score matched estimates). Two sensitivity analyses extended the primary analysis: (1) adding indirect comparisons: for studies reporting outcomes relative to a shared no-epinephrine reference group, indirect early-versus-late ORs were calculated as the ratio of early-category to late-category ORs (Bucher method), marked with † in forest plots; and (2) adding unadjusted (crude) estimates. Adjusted ORs, propensity-score matched RRs, and crude ORs were combined on the log scale in these sensitivity analyses, with approximate OR–RR equivalence assumed given low event rates (<5% for neurological outcome and survival); this approximation is less secure in the shockable subgroup, where favorable outcomes can exceed 5%, so the corresponding pooled magnitudes warrant additional caution.

**Time–response meta-analysis.** For studies with ≥3 timing categories, two-stage dose–response meta-analysis was performed, where the “dose” refers to time-to-epinephrine as a continuous exposure variable. First, a two-stage linear dose–response meta-analysis estimated pooled per-minute ORs from categorical data using the Greenland–Longnecker method [39]. Second, non-linear modeling used 3-knot restricted cubic splines (knots at 5, 15, and 30 min, selected to approximate the 10th, 50th, and 90th percentiles of the pooled exposure distribution). Study-specific spline coefficients were estimated using generalized least squares and pooled using multivariate random-effects meta-analysis (REML). Given that time reference points varied substantially across studies (see below), these curves should be interpreted as exploratory approximations of the overall time–response shape rather than precise predictions at specific time points. Sensitivity analyses explored alternative knot placements (Figure S1). Data extraction details are provided in Table S3.

**Time reference points.** Studies did not share a common origin for “time-to-epinephrine”. Four origins were used: the emergency call, initiation of CPR or EMS patient contact, EMS arrival on scene and, in one study, the first defibrillation attempt. These intervals are nested rather than interchangeable—call-to-epinephrine contains the dispatch and response intervals that arrival-to-epinephrine excludes—so the same number of minutes does not describe the same clinical situation in different studies. We therefore did not rescale or align the time origins, and we treat every pooled estimate as a relative gradient (the change in odds associated with one additional minute of delay or with a study’s own early-versus-late contrast) rather than as the outcome expected at an absolute time on a shared clock. Each study’s origin is recorded in Table 1 and Table S3. To quantify how much this heterogeneity matters, we repeated the per-minute and categorical analyses after restricting them to studies sharing a single origin, and we tested the time origin as a subgroup variable (Table S5).

**Subgroup and sensitivity analyses.** All analyses were stratified by rhythm. Sensitivity analyses included restriction to direct comparisons, adjusted estimates only, while excluding crude ORs, RRs, non-overlapping registries, restriction to a common time origin (Table S5), and studies with critical risk of bias. Leave-one-out sensitivity analysis was performed for the primary per-minute analysis. Influence diagnostics (studentized residuals, DFFITS, Cook’s distance and hat values) were computed for the same analysis, and small-study effects were examined by visual inspection of funnel plots (Figures S2 and S3); formal tests of funnel-plot asymmetry were not performed, because fewer than ten studies contributed to each pooled analysis. Heterogeneity was quantified using I^2^ and Cochran’s Q [40]; for random-effects estimates with substantial heterogeneity, 95% prediction intervals (Higgins–Thompson–Spiegelhalter method) were also calculated to express the range of true effects expected in a future study. E-values were additionally computed for the principal pooled and single-study estimates to quantify the strength of unmeasured confounding that would be required to explain them away (EValue R package; Table S6). An E-value states the strength of confounding that would be required for an association to be spurious; it does not indicate whether confounding of that strength is plausible. In this setting it is, because epinephrine timing is recorded on the same resuscitation timeline that determines survival, so resuscitation time bias alone can generate associations of the size observed. Large E-values here should therefore not be read as evidence of robustness.

**Outcome definitions.** Favorable neurological outcome was defined as Cerebral Performance Category (CPC) 1–2 or modified Rankin Scale (mRS) ≤ 3. These thresholds are considered broadly equivalent for defining functional independence after cardiac arrest [24,41,42]. In the categorical analysis, all contributing studies used CPC 1–2; in the per-minute neurological analysis, one of three studies (Hansen 2018) [10] used mRS ≤ 3, and a sensitivity analysis excluding this study was performed.

**Overlap management.** Many of the Japanese studies drew on the same nationwide Fire and Disaster Management Agency (FDMA) registry over overlapping calendar years, so the same patients could be counted in more than one study. To avoid double-counting, whenever two or more studies covered the same registry and overlapping period we included only one of them in a given meta-analysis, selecting, in order of priority, the study with (1) the most relevant timing-and-outcome analysis, (2) the most rigorous confounding adjustment, and (3) the largest sample size; the remaining overlapping studies were not pooled in that analysis. Table S4 maps every study to its registry and data years and documents each decision. Concretely, seven studies draw on the FDMA registry over windows that overlap to differing degrees; the conservative analysis retained Fukuda 2021 [9] (2011–2017) and Kawakami 2024 [14] (2011–2020, shockable only) as the largest and most recent series and excluded Goto 2013 [11], Fukuda 2016 [33], Tanaka 2016 [26], Funada 2018 [12] and Otani 2026 [17] as subsumed. The same rule was applied outside Japan: Hansen 2018 [10] and Okubo 2021 [13] both analyze the Resuscitation Outcomes Consortium registry, so Hansen 2018 [10] was dropped from the non-overlapping per-minute analysis, and Enzan 2022 [16] was dropped as subsumed by Sakamoto 2025 [20] within JAAM-OHCA. To confirm that any residual overlap did not drive the results, we additionally ran the most conservative possible version of the analysis, retaining only studies drawn from entirely non-overlapping registry periods (one study per FDMA period), which yielded consistent estimates (Table S7).

**Certainty of evidence.** Certainty of evidence was assessed using the Grading of Recommendations, Assessment, Development and Evaluations (GRADE) framework [43]: evidence from observational studies started at “low” certainty and was rated across five domains (risk of bias, inconsistency, indirectness, imprecision, and publication bias), with potential upgrading for large effects, dose–response gradients, or plausible residual confounding that would bias toward the null. We did not invoke any of these upgrades, because the large effects and apparent dose–response observed here are plausibly generated by resuscitation time bias rather than a causal timing effect; residual confounding that, in this setting, biases away from rather than toward the null. Full methodological details are provided in the Supplementary Methods.

All analyses were performed using R version 4.5.2 with the meta, metafor, mvmeta, and dosresmeta packages.

## 3. Results

### 3.1. Study Selection and Characteristics

**Figure 1:**
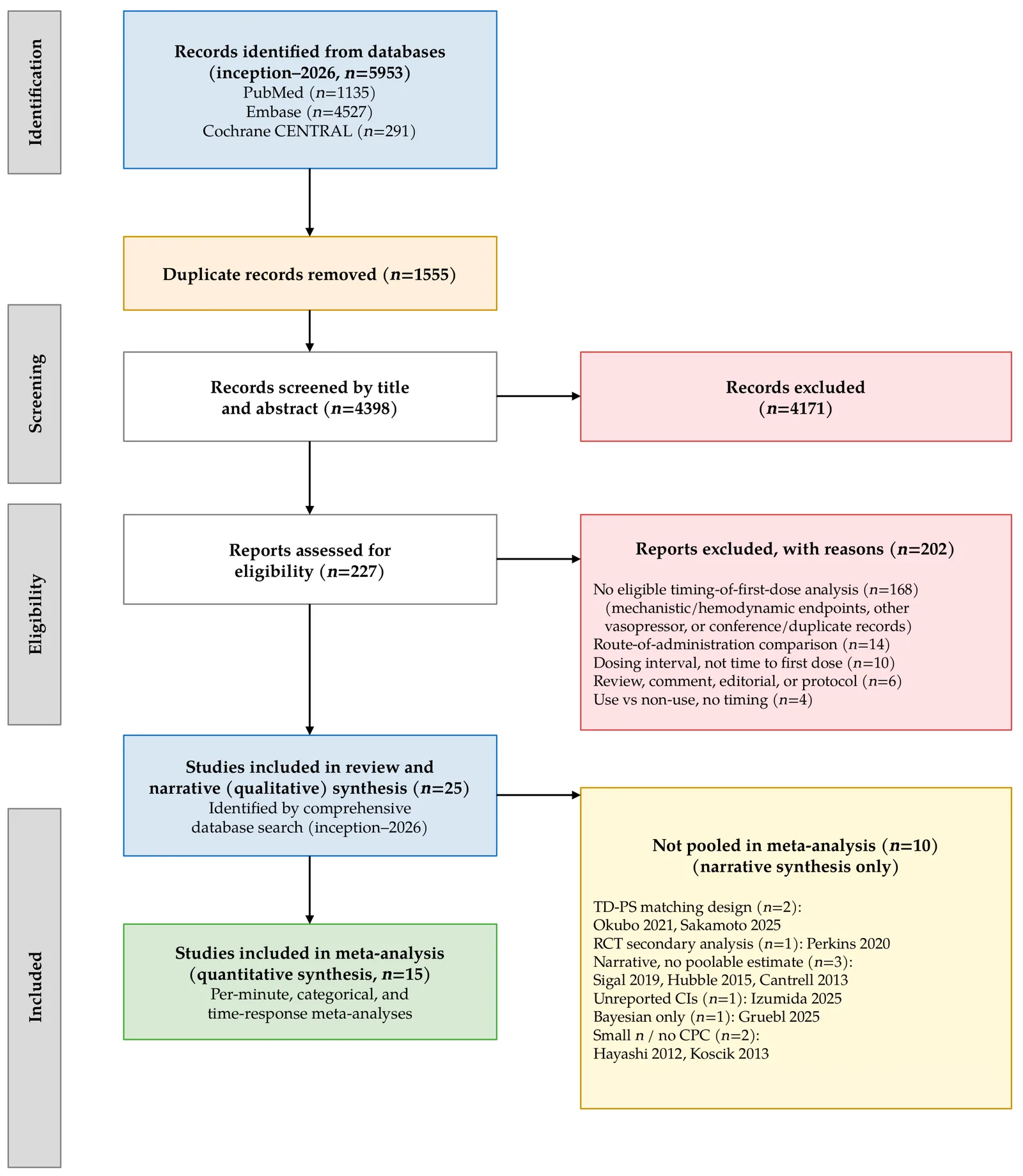
PRISMA 2020 flow diagram. Studies listed in the flow diagram are cited as follows: Okubo 2021 [13], Sakamoto 2025 [20], Perkins 2020 [38], Sigal 2019 [37], Hubble 2015 [32], Cantrell 2013 [30], Izumida 2025 [19], Gruebl 2025 [21], Hayashi 2012 [27], and Koscik 2013 [29].

The search of PubMed, Embase, and CENTRAL from inception through March 15, 2026 identified 5953 records (PubMed 1135; Embase 4527; CENTRAL 291). After removal of 1555 duplicate records (deduplication by normalized title and digital object identifier), 4398 records were screened by title and abstract. Following a reproducible relevance screen (records mentioning cardiac arrest together with epinephrine, adrenaline, or a vasopressor, with exclusion of pediatric, in-hospital, animal, sepsis, trauma, and burn records; Supplementary Methods), 227 reports were assessed for eligibility (Figure 1). Of these, 202 were excluded: 168 did not report an eligible analysis of the timing of the first epinephrine dose (including mechanistic or hemodynamic endpoints, other vasopressors, and conference or duplicate records), 14 compared routes of administration, 10 examined epinephrine dosing interval rather than time-to-first-dose, six were reviews, comments, editorials, or protocols, and four compared epinephrine use versus non-use without a timing analysis. Twenty-five studies met inclusion criteria. Fifteen studies contributed to at least one meta-analysis; the remainder were included in narrative synthesis due to methodological incompatibility (Bayesian analysis only, time-dependent propensity-score designs, unreported CIs, or return-of-spontaneous-circulation-only data from overlapping cohorts). The 25 studies reported over 1,200,000 patient records (counting overlapping registry cohorts more than once, so not all unique patients) across seven countries, with 14 studies (56%) from Japan, many using overlapping Fire and Disaster Management Agency (FDMA) registry data (Table S4). Key contributing cohorts included Fukuda 2021 [9] (N = 119,946; per-minute and categorical analyses), Kawakami 2024 [14] (N = 20,905; defibrillation-to-epinephrine analysis), Otani 2026 [17] (N = 31,670; time–response modeling), Okada 2025 [18] (N = 23,051; multinational cohort from Korea and Singapore), Izumida 2025 [19] (N = 184,731; largest single timing study, not pooled due to unreported CIs), and Sagisaka 2017 [35] (N = 11,876; categorical neurological and survival analyses). Time reference points varied substantially across studies: emergency-call-to-epinephrine (*n* = 8), CPR-initiation- or EMS-contact-to-epinephrine (*n* = 5), EMS-arrival-to-epinephrine (*n* = 4), and defibrillation-to-epinephrine (*n* = 1). These origins are not interchangeable: ten minutes measured from the emergency call includes the dispatch and response intervals and typically corresponds to only a few minutes of EMS patient contact, whereas ten minutes measured from EMS arrival describes a patient who has already been in arrest considerably longer. Pooled estimates should therefore be read as relative gradients across each study’s own timing distribution, not as effects attached to an absolute time on a shared clock, and this heterogeneity is a key limitation of the pooled estimates. Table 1 presents study characteristics; Table 2, Table 3, Table 4, Table 5, Table 6 and Table 7 summarize all meta-analysis results.

### 3.2. Per-Minute Meta-Analysis

For **non-shockable rhythms**, four studies (Ewy 2015 [31], Hansen 2018 [10], Homma 2019 [36], Enzan 2022 [16]; N = 40,449) yielded a pooled OR of 0.958 per minute (95% CI 0.949–0.968; I^2^ = 0%; 95% prediction interval 0.94–0.98), indicating a 4.2% reduction in survival odds per minute of delay across heterogeneous time references (dispatch-to-epinephrine, arrival-to-epinephrine, and call-to-epinephrine). Three studies (N = 31,823) reported a consistent per-minute reduction in neurological outcome (OR 0.953, 95% CI 0.929–0.978; I^2^ = 0%). Leave-one-out analysis indicated stability (Table S8 and Figure S4), no study was flagged as influential by the studentized-residual, DFFITS or hat diagnostics (Figure S5), and the individual study estimates contributing to this pooled OR are shown in the forest plot in Figure S6. This estimate is descriptive and system-level: it summarizes how outcomes differ across patients who received epinephrine at different points in their own resuscitation, and it is not an estimate of the effect that would follow from moving an individual patient’s dose earlier. Restricting the analysis to studies sharing a single time origin left it essentially unchanged (call- or dispatch-anchored studies, k = 3: OR 0.957, 95% CI 0.945–0.969, I^2^ = 0%; strictly EMS-call-anchored, k = 2: OR 0.957, 95% CI 0.945–0.970; the single EMS-arrival-anchored study, Hansen 2018 [10]: OR 0.96, 95% CI 0.95–0.98), and the time origin explained none of the between-study variation (subgroup *p* = 0.75; Table S5).

For **shockable rhythms**, only two studies (N = 2169) were available, yielding inconclusive results (OR 0.980, 95% CI 0.904–1.061; I^2^ = 93.7%) due to opposing effect directions: Ewy 2015 [31] (US) reported OR 0.94 (0.91–0.97) favoring early epinephrine, while Homma 2019 [36] (Japan) reported OR 1.02 (0.99–1.04) suggesting no benefit; this discrepancy is potentially attributable to different time references and EMS systems. For **overall** rhythms (not rhythm-stratified), two studies (Fukuda 2021 [9], N = 119,946; Hubble 2017 [34], N = 2100) yielded OR 0.910 per minute (95% CI 0.900–0.919; I^2^ = 0%), heavily weighted by Fukuda 2021 [9].

### 3.3. Categorical Early-Versus-Late Meta-Analysis

**Figure 2:**
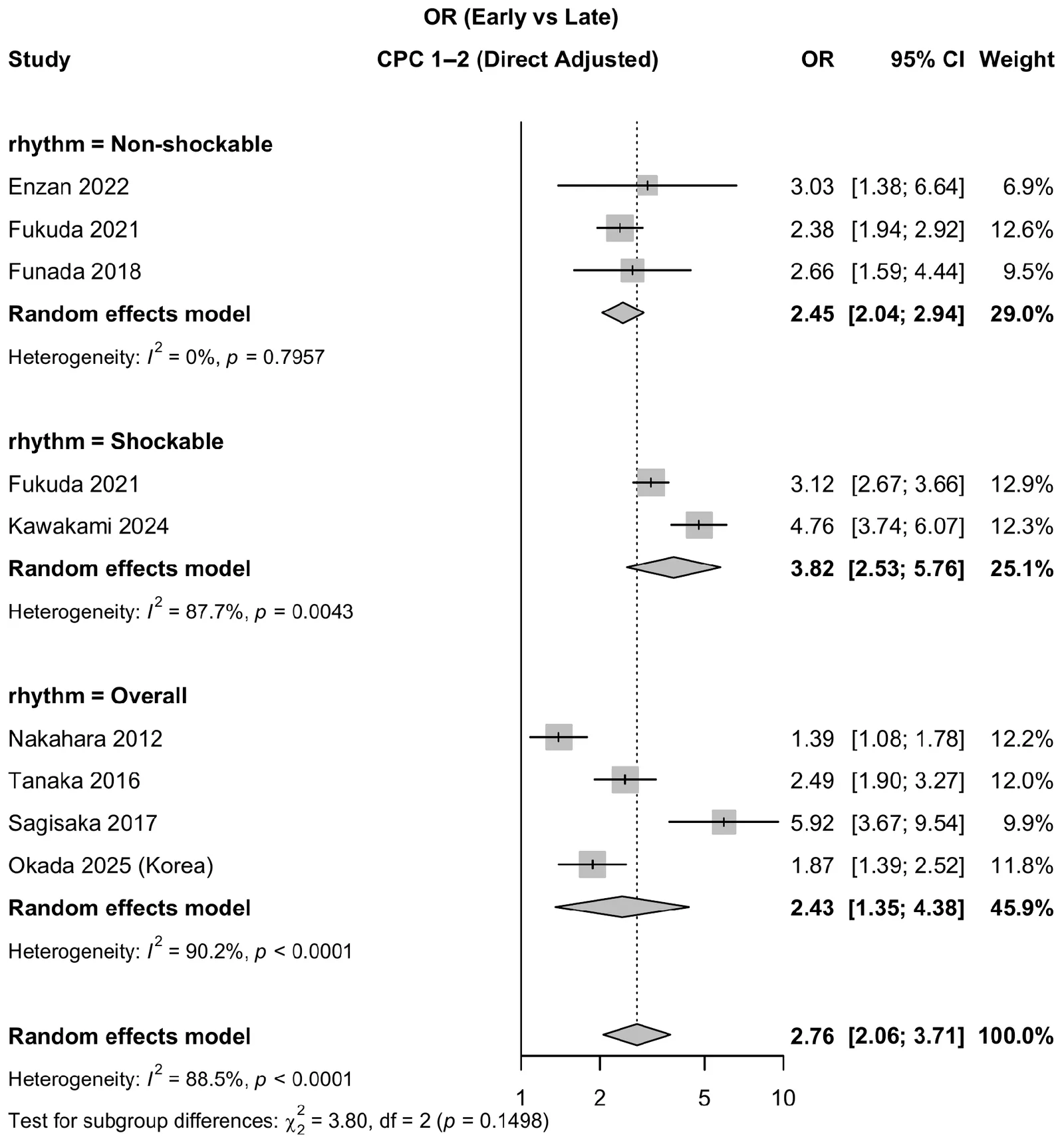

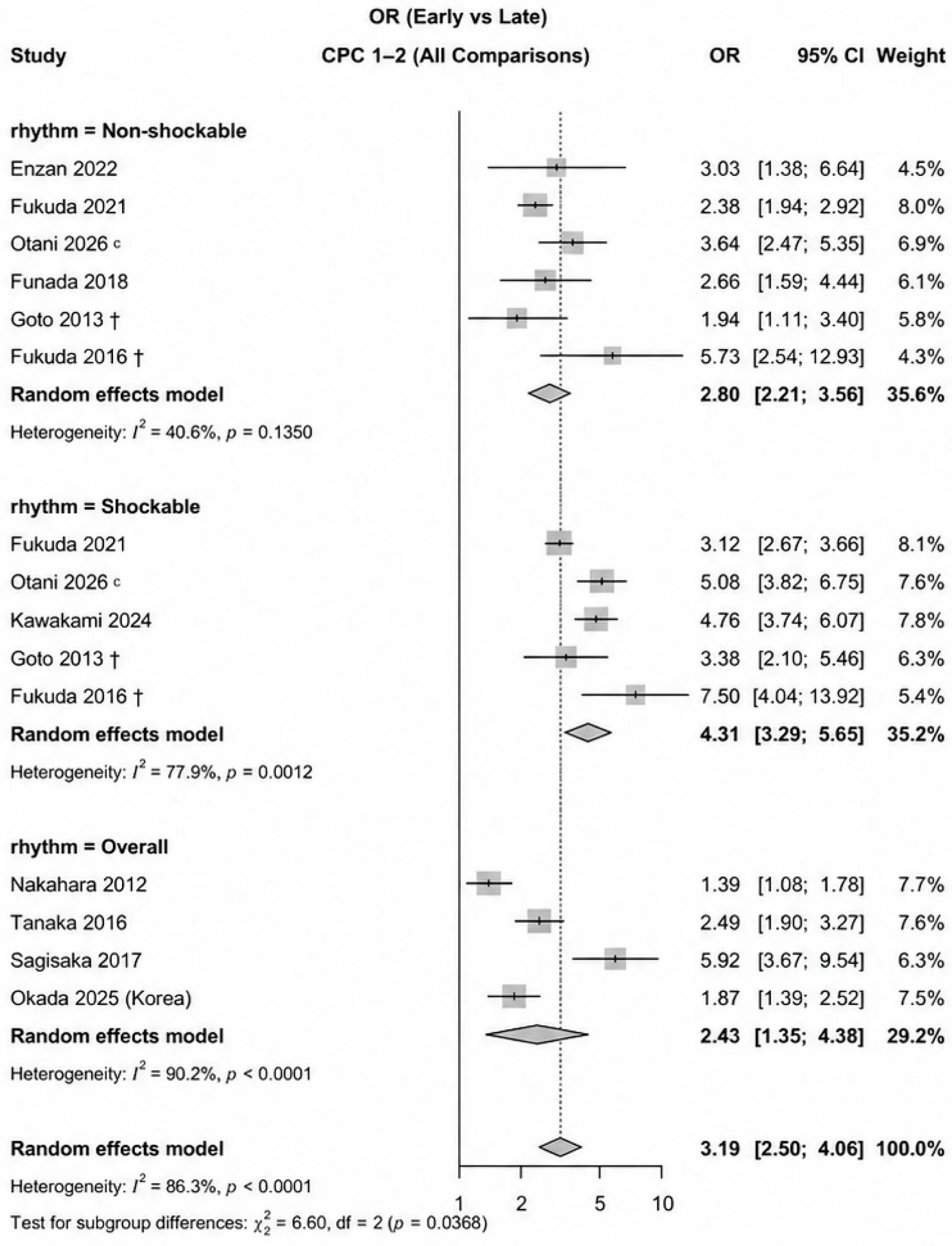
Forest plot: Categorical early vs. late OR for neurological outcome (CPC 1–2). In each panel, the solid vertical line indicates no effect (OR = 1) and the dotted vertical line indicates the overall pooled random-effects estimate. Primary analysis (direct adjusted comparisons, k = 9) and comprehensive sensitivity analysis (including indirect comparisons (†) and crude estimates (^c^), k = 15) are shown. Indirect comparisons are derived from OR ratios via shared no-epinephrine reference groups. Contributing time origins differ: emergency call (Enzan 2022 [16], Fukuda 2021 [9], Tanaka 2016 [26], Sagisaka 2017 [35], Otani 2026 [17], Fukuda 2016 [33]), CPR initiation (Nakahara 2012 [28], Funada 2018 [12], Goto 2013 [11]), EMS arrival (Okada 2025 [18]) and first defibrillation (Kawakami 2024 [14]), so the pooled estimates are relative gradients rather than effects at a common clock time; a sensitivity analysis restricted to a single origin is given in Table S5. “Early” versus “late” was defined within each study using its own timing dichotomization or most extreme timing categories rather than a single common threshold (study-specific definitions in Table 1 and Table S3); these heterogeneous thresholds, together with differing time reference points, contribute to the between-study heterogeneity.

**Primary analysis (direct adjusted comparisons).** Nine study–outcome combinations from eight studies using direct early-versus-late comparisons with adjusted estimates contributed to the primary categorical neurological outcome analysis (Figure 2). The pooled OR was 2.76 (95% CI 2.06–3.71; I^2^ = 88.5%; 95% prediction interval 0.98–7.79), favoring early epinephrine. When stratified by rhythm, the non-shockable subgroup (k = 3) showed OR 2.45 (95% CI 2.04–2.94; I^2^ = 0%), the shockable subgroup (k = 2) OR 3.81 (95% CI 2.52–5.77; I^2^ = 88%), and the overall mixed-rhythm subgroup (Nakahara 2012 [28], Tanaka 2016 [26], Sagisaka 2017 [35], Okada 2025 [18]; k = 4) OR 2.43 (95% CI 1.35–4.38; I^2^ = 90%). The difference between shockable and non-shockable subgroups was not statistically significant in this primary analysis (*p* = 0.053).

**Comprehensive sensitivity analysis.** When indirect comparisons (Goto 2013 [11], Fukuda 2016 [33]; derived from shared no-epinephrine reference groups), an additional direct adjusted estimate (Sagisaka 2017 [35]), and unadjusted estimates (Otani 2026 [17]) were added, 15 study–outcome combinations from 11 studies contributed (Figure 2). The pooled OR was 3.19 (95% CI 2.50–4.06; I^2^ = 86.3%; 95% prediction interval 1.22–8.31). By rhythm:

**Shockable** (k = 5): OR 4.31 (95% CI 3.29–5.65; I^2^ = 77.9%; 95% PI 1.72–10.81).

**Non-shockable** (k = 6): OR 2.80 (95% CI 2.21–3.56; I^2^ = 40.6%; 95% PI 1.54–5.10).

**Overall** (k = 4): OR 2.43 (95% CI 1.35–4.38; I^2^ = 90.2%).

The shockable-versus-non-shockable difference was statistically significant in this comprehensive analysis (*p* = 0.020; three-group test *p* = 0.038) but not in the primary analysis restricted to direct adjusted comparisons (*p* = 0.053; three-group test *p* = 0.15); the rhythm contrast thus emerges only when lower-quality indirect and unadjusted comparisons are added and should be regarded as suggestive rather than established. The indirect ORs from Fukuda 2016 [33] were also large (e.g., 7.50 for shockable rhythm), as was the adjusted estimate from Sagisaka 2017 [35] (5.92 for the overall cohort); such magnitudes are implausible for a causal timing effect and most plausibly reflect residual confounding together with the treatment-versus-no-treatment signal embedded in the shared no-epinephrine reference (see Discussion). All sensitivity analyses maintained statistical significance and a consistent direction (OR range 2.55–3.78 across all sensitivity analyses, including the time-origin restrictions below; Tables S7 and S5), indicating a consistent direction despite substantial variation in magnitude.

**Sensitivity to the time origin.** Restricting the primary categorical analysis to the five estimates anchored on the emergency call gave a pooled OR of 3.07 (95% CI 2.28–4.13; I^2^ = 72.0%), and excluding the single defibrillation-anchored estimate (Kawakami 2024 [14]) gave 2.55 (95% CI 1.91–3.40; I^2^ = 84.6%), against 2.76 (2.06–3.71; I^2^ = 88.5%) for the full primary set. Restricting to a common origin therefore reduced heterogeneity without removing it and moved the point estimate by roughly 20% in either direction depending on which origin was retained; time origin was a statistically significant source of between-study variation when tested across all four origins (*p* < 0.001), although that test rests on two single-study subgroups. In the comprehensive analysis the call-anchored subset (k = 9) gave OR 3.78 (95% CI 2.89–4.95; I^2^ = 78.8%), and within this time-homogeneous subset the difference between rhythms was no longer apparent (shockable k = 3, OR 4.61, 95% CI 2.86–7.44; non-shockable k = 4, OR 3.16, 95% CI 2.22–4.48; between-rhythm *p* = 0.46), compared with *p* = 0.020 in the full comprehensive set. Full results are given in Table S5.

Six study–outcome combinations contributed categorical survival data, yielding a pooled OR of 2.08 (95% CI 1.48–2.93; I^2^ = 92.4%; 95% prediction interval 0.61–7.05) favoring early epinephrine, albeit with considerable heterogeneity driven partly by the large adjusted estimate from Sagisaka 2017 [35].

### 3.4. Time–Response Meta-Analysis (Exploratory)

**Figure 3:**
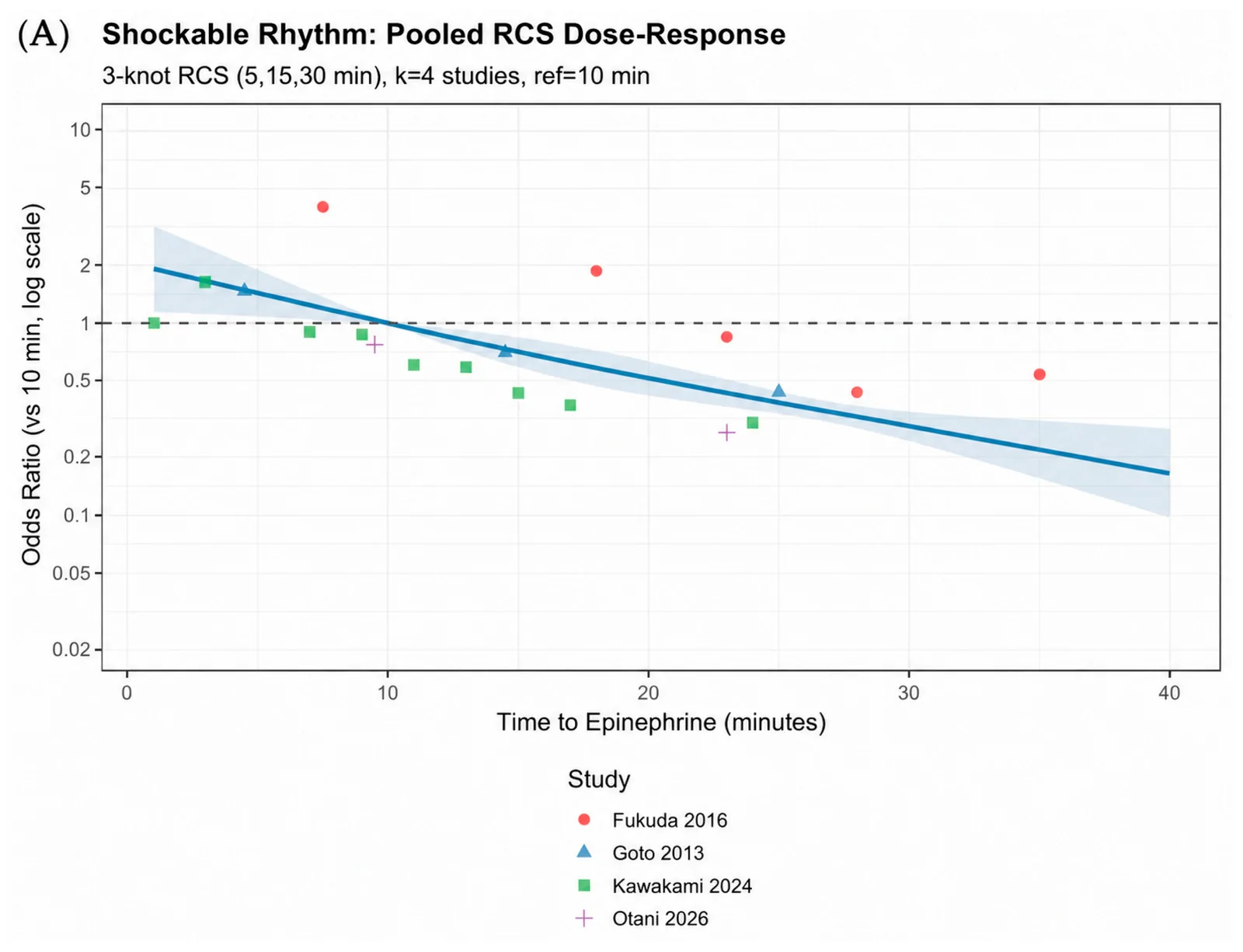

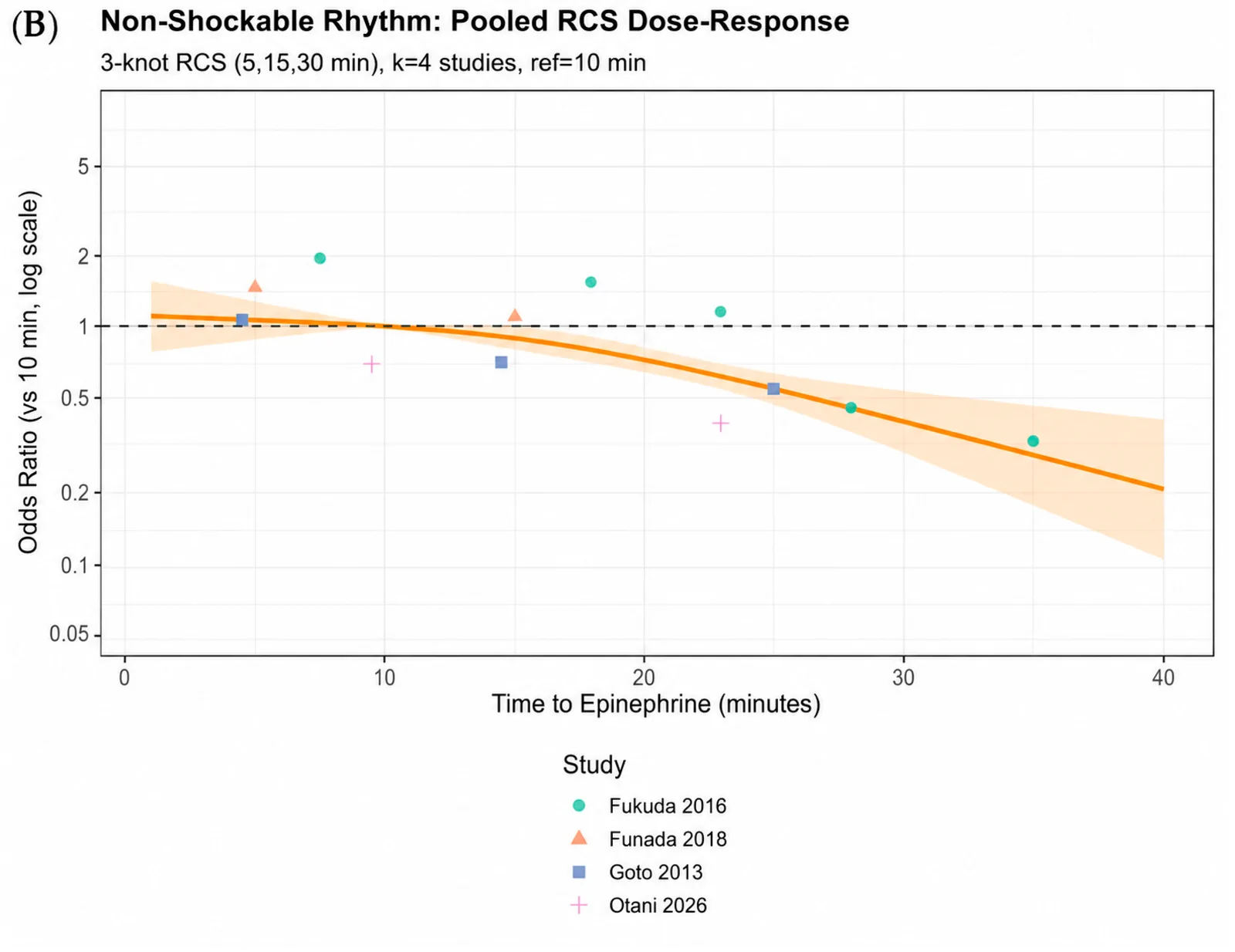
Pooled restricted cubic spline time–response curves (exploratory) for CPC 1–2, centered at 10 min. Splines use three knots, placed at 5, 15, and 30 min: (**A**) shockable rhythm, (**B**) non-shockable rhythm, with individual study data points overlaid. Panel (**A**) pools four studies (Goto 2013 [11], Fukuda 2016 [33], Kawakami 2024 [14], Otani 2026 [17]); Panel (**B**) pools four studies (Goto 2013 [11], Fukuda 2016 [33], Funada 2018 [12], Otani 2026 [17]). The time reference point differs across contributing studies, being CPR initiation (Goto 2013 [11], Funada 2018 [12]), the emergency call (Fukuda 2016 [33], Otani 2026 [17]) or first defibrillation (Kawakami 2024 [14]), so the x-axis does not represent a single common clock and times are not comparable across studies. Shaded bands are 95% confidence bands; they widen markedly towards both extremes of the range, where few studies contribute data. Between-study heterogeneity is very high (I^2^ = 94.9% for panel (**A**) and 79.6% for panel (**B**)). These curves are exploratory and hypothesis-generating: they describe the shape of the pooled association and must not be used to identify an optimal or safe time window.

The pooled three-knot RCS curves (Figure 3) showed decreasing time–response relationships for both rhythms, centered at 10 min (OR = 1.00). These curves are exploratory and hypothesis-generating. Each rests on four heterogeneous observational studies that do not share a time origin (Table S3), between-study heterogeneity is very high (I^2^ = 80–95%), and the confidence bands widen sharply towards both extremes of the modeled range, where few studies contribute data. They should not be used to identify an optimal time window or a period during which delay is safe; the values below describe the shape of the pooled curve and are not thresholds:

**Shockable rhythm** (k = 4: Goto 2013 [11], Fukuda 2016 [33], Kawakami 2024 [14], Otani 2026 [17]): The curve was approximately log-linear, with OR 1.44 (95% CI 1.09–1.90) at 5 min, 0.52 (0.42–0.64) at 20 min, and 0.29 (0.24–0.35) at 30 min (I^2^ = 94.9%).

**Non-shockable rhythm** (k = 4: Goto 2013 [11], Fukuda 2016 [33], Funada 2018 [12], Otani 2026 [17]): The curve showed a relatively flat region from 5 to 15 min (OR 1.06 at 5 min and 0.89 at 15 min, neither significantly different from reference), followed by steeper decline: OR 0.73 (0.64–0.82) at 20 min and 0.40 (0.30–0.54) at 30 min (I^2^ = 79.6%).

The shockable curve was steeper than the non-shockable curve throughout, with both converging to similarly low ORs by 30–35 min (Figure S7). The two-stage linear dose–response meta-analysis yielded pooled ORs per minute of 0.941 (0.919–0.963) for shockable and 0.971 (0.957–0.985) for non-shockable rhythm (between-group *p* = 0.067).

### 3.5. Risk of Bias and Certainty of Evidence

ROBINS-I assessment (Table S9) rated three studies (12%) at moderate overall risk of bias (Okubo 2021 [13], Perkins 2020 [38], Sakamoto 2025 [20]), all employing time-dependent propensity-score methods or a randomized design; 21 studies (84%) were rated at serious risk, and one (4%; Koscik 2013 [29]) at critical risk. The predominant concern was confounding (domain 1): standard multivariable regression, used by most studies, cannot fully address the time-dependent confounding and resuscitation time bias inherent in epinephrine timing analyses. No study controlled for CPR quality or vascular access difficulty. GRADE certainty was “very low” for all eight assessed outcomes (Table S10), reflecting the observational evidence base and serious risk of bias due to residual confounding. Formal small-study and publication-bias testing was constrained because individual pooled analyses included fewer studies than the conventional threshold for reliable funnel-plot assessment, so publication bias cannot be excluded; the corresponding funnel plots (Figures S2 and S3) are therefore presented for visual inspection only.

### 3.6. Narrative Synthesis

The unpooled studies matter because several of them address questions the pooled analyses cannot. Three questions must be kept apart. First, does epinephrine work at all? Only PARAMEDIC2 [4] answers this, by randomization, and the answer is that it improves 30-day survival without improving neurological outcome. Second, is the effect of epinephrine versus placebo modified by how early it is given? The PARAMEDIC2 timing analysis [38] answers this, again under randomization, and finds no such modification. Third, among patients who all received epinephrine, do those treated earlier fare better? This is the question our pooled estimates address, no randomized evidence answers it, and it is the only one of the three exposed to resuscitation time bias. The time-dependent propensity-score studies [13,20] address this third question with designs better suited to time-varying confounding than the studies we pooled, which is why their results are reported here rather than combined with ours. Ten studies were not pooled. Okubo 2021 [13] and Sakamoto 2025 [20] used time-dependent propensity-score matching and reported consistent time–response patterns favoring earlier epinephrine; Okubo noted paradoxically increased ROSC rates with delay, likely reflecting immortal time bias. Perkins 2020 [38], a prespecified secondary analysis of the PARAMEDIC2 randomized controlled trial, examined whether the treatment effect of epinephrine versus placebo was modified by time-to-drug-administration and found no significant timing interaction for survival (interaction OR 0.98/min, 95% CI 0.94–1.03) or neurological outcome (interaction OR 0.98/min, 95% CI 0.93–1.03); this addresses a related but distinct question (whether the drug effect varies with time) from the observational question of early-versus-late timing among treated patients. Sigal 2019 [37], a registry analysis restricted to shockable rhythm, found that survivors received adrenaline earlier than non-survivors (median 5.0 vs. 7.0 min, *p* = 0.022) but observed no significant association between timing and neurological outcome among survivors, a null finding that tempers the large timing effects observed elsewhere for shockable rhythm. Two North Carolina EMS cohorts reported ROSC as a function of vasopressor timing: Hubble 2015 [32] found a 4% per-minute decline in the odds of ROSC (OR 0.96, 95% CI 0.93–0.98), and Cantrell 2013 [30] reported higher ROSC with administration ≤10 versus >10 min (adjusted OR 1.91, 95% CI 1.01–3.63); both overlap with the Hubble 2017 cohort [34] and were not pooled to avoid double-counting and because ROSC was a secondary outcome. The remaining studies were excluded from pooling due to unreported CIs [19], Bayesian-only analysis [21], or small per-rhythm subgroups [27].

## 4. Discussion

This systematic review and time–response meta-analysis of 25 studies identified a consistent inverse association between time-to-epinephrine and outcomes. However, the reliability of this evidence varies substantially across analyses, and, importantly, all results must be interpreted in the context of very low GRADE certainty, heterogeneous time reference points, and the fundamental challenge of causal inference from observational timing data, in which the association is shaped as much by which patients receive early epinephrine as by the timing itself.

### 4.1. Principal Findings and What This Review Adds

Compared with the prior meta-analysis by Ran et al. [15], this synthesis differs in what it searched, what it pooled, and how it appraised what it found. It adds a from-inception three-database search extended to March 2026, contributing cohorts published since that review, among them the five first reported in 2025 [17,18,19,20,21]. It provides three forms of evidence not previously quantified for epinephrine timing: a per-minute (continuous) dose–response estimate, rhythm-stratified pooled estimates, and pooled restricted cubic spline modeling of the time–response shape. It adds an appraisal layer the earlier review did not have: ROBINS-I risk of bias, GRADE certainty, explicit registry-overlap management for the Japanese nationwide cohorts, E-value quantification of the confounding required to explain the results away, and sensitivity analysis restricted to a common time origin. These additions differ in reliability and clinical significance, and none of them overcomes the core problem of confounding; if anything, the appraisal layer makes that problem more visible than it was before.

**Per-minute quantification for non-shockable rhythm.** The pooled per-minute OR of 0.958 (I^2^ = 0%), consistent across four studies spanning US, US/Canada, and Japanese cohorts with different time references, is the most internally consistent finding of this review. Its target of inference should be stated plainly: it is a descriptive, system-level association summarizing how outcomes vary across patients treated at different points in their own resuscitation, not an estimate of the effect of intervening to give a particular patient epinephrine one minute earlier. It is also robust in a narrow sense only—it does not depend on which studies or which time origins are included (Table S5)—and that is a statement about internal consistency, not about freedom from bias. The same resuscitation time bias operates in all four contributing studies and would be expected to produce agreement between them rather than to disturb it. Current guidelines recommend epinephrine “as soon as feasible” for non-shockable rhythms [2,3,5,6] but do not quantify the cost of delay. This estimate provides an approximation: each additional minute is associated with approximately 4.2% lower survival odds, and a 5 min delay would translate to a ~20% reduction (0.958^5^ = 0.81). However, the magnitude of this estimate likely reflects a combination of true timing effects and residual confounding from resuscitation time bias [22], and the I^2^ = 0% pertains to between-study consistency rather than the certainty of the causal effect and, with only four studies, carries limited power to detect heterogeneity. Its 95% prediction interval (0.94–0.98) also excludes the null, unlike the wider categorical prediction intervals discussed below. Nonetheless, this level of specificity, not available from prior meta-analyses, may be informative for evaluating time-to-epinephrine as a potential EMS quality indicator, provided it is used to track system performance rather than to predict the benefit an individual patient would derive from earlier treatment.

**Rhythm-specific pooled estimates.** The apparent effect was larger for shockable (OR 4.31, I^2^ = 78%) than non-shockable rhythms (OR 2.80, I^2^ = 41%), a rhythm-stratified comparison not reported in prior meta-analyses, but this difference reached statistical significance only in the comprehensive analysis (*p* = 0.020), not in the primary analysis restricted to direct adjusted comparisons (*p* = 0.053); it is significant only when the lower-quality indirect and unadjusted comparisons are included. The shockable estimate is also the least reliable subgroup, depending heavily on indirect comparisons and unadjusted estimates. The new time-origin restriction sharpens this caution: within the comprehensive set restricted to call-anchored studies, the shockable-versus-non-shockable difference was no longer apparent (*p* = 0.46; Table S5). The rhythm contrast therefore depends both on including lower-quality comparisons and on pooling across non-equivalent time origins and should be treated as hypothesis-generating rather than as an established difference. What is consistent is the direction of effect: every sensitivity analysis favored early epinephrine (OR range 2.55–3.78). Moreover, the 95% prediction intervals for the categorical estimates are wide and, for the primary direct adjusted analysis, include the null (0.98–7.79), so the substantial between-study heterogeneity leaves open the possibility of little or no effect in a future setting. Directional consistency, however, does not address the magnitude inflation discussed below, to which all of these estimates are subject.

**Dose–response curve shapes (exploratory).** The pooled RCS curves suggest a log-linear decline for shockable rhythm and a flat early region (5–15 min) followed by a steeper decline for non-shockable rhythms. These shapes are hypothesis-generating only. Each curve rests on four observational studies with different time origins and very high heterogeneity (I^2^ = 80–95%), and the uncertainty widens markedly at both ends of the modeled range. The apparent flat region for non-shockable rhythms is most plausibly an artifact of the differing contributing study sets and time references rather than a physiologically meaningful “safe window”, and reading a permissible delay off these curves would be a misuse of them: a curve pooled across studies that start their clocks at different events cannot locate a threshold on any single clock.

Overall, the main addition relative to Ran et al. [15] is the per-minute quantification and the explicit appraisal of confounding; only the non-shockable per-minute estimate achieves precision and between-study consistency sufficient to cautiously inform practice, and even that estimate cannot be interpreted causally.

### 4.2. Confounding and Causal Inference

These associations should not be interpreted causally. As Andersen et al. [22] have described, a fundamental “resuscitation time bias” operates in observational cardiac arrest timing studies: patients who have not yet received treatment are, by definition, those who have not yet achieved ROSC, creating inherent selection toward worse prognosis with longer time-to-treatment. This bias cannot be fully addressed by standard multivariable regression. Patients receiving later epinephrine may also differ systematically in ways unmeasured by most studies (e.g., difficult vascular access, poor CPR quality, provider experience). Furthermore, for shockable rhythms specifically, early defibrillation is the priority intervention, and early receipt of epinephrine may simply be a marker of rapid access to advanced life support rather than a causal driver of outcomes. Even in ALS randomized trials, patients who receive early placebo tend to have better outcomes because early drug administration reflects the presence of an advanced resuscitation team [4,38]. The magnitude of several pooled inputs illustrates this concern: an adjusted OR of 5.92 (Sagisaka 2017 [35]) or an indirect OR of 7.50 (Fukuda 2016 [33]) would imply that earlier epinephrine multiplies the odds of intact survival six- to seven-fold, far exceeding the effect of any established resuscitation intervention and biologically implausible as a pure timing effect. A formal E-value sensitivity analysis (Table S6) quantifies this: fully explaining away the pooled associations would require unmeasured confounding associated with both epinephrine timing and outcome at a risk ratio of approximately 3.6–5.0 for the primary and non-shockable estimates, rising to 8.1 for the shockable estimate and 14.5 for the indirect comparison (Fukuda 2016 [33]). Although large E-values are conventionally read as evidence of robustness, confounding of this magnitude is plausible in this setting, because epinephrine timing is recorded along the same resuscitation timeline that governs survival, so resuscitation time bias alone can generate associations of the observed size. Such values are better understood as a quantitative signature of residual confounding and shared-reference-group artifacts than as causal estimates, and they are a principal reason we frame this synthesis as descriptive rather than causal.

The only randomized evidence, a PARAMEDIC2 secondary analysis [38], did not find a significant timing interaction for survival or neurological outcome. Although that analysis tested a related but distinct question (whether the drug effect of epinephrine vs. placebo was modified by time), the null finding for timing interaction in a randomized context should temper the strength of inference from observational timing associations.

Most included studies adjusted for Utstein covariates, but none accounted for CPR quality or vascular access difficulty. The three studies employing time-dependent propensity-score methods [13,20,38], which are better suited to address time-varying confounding, were not pooled quantitatively, further limiting the strength of causal inference from the pooled estimates.

Substantial heterogeneity (I^2^ = 77–96%) in categorical and dose–response analyses reflects different time reference points, reference groups, effect measures, and EMS systems. Heterogeneous time references are particularly problematic for shockable rhythms, where defibrillation-to-epinephrine [14] and call-to-epinephrine intervals represent non-equivalent scales, a major contributor to the extreme heterogeneity (I^2^ = 95%) in the shockable RCS analysis, alongside differences in adjustment methods and population characteristics. The time-origin analyses quantify this: restricting to a single origin lowered I^2^ in the primary categorical analysis from 88.5% to 72.0% and in the comprehensive analysis from 86.3% to 78.8% (Table S5), so non-equivalent time origins are a substantial but not the sole source of the heterogeneity. This bears directly on how the pooled numbers should be read. A random-effects summary is an average across settings, not a value any particular EMS system should expect, and the prediction intervals express that distinction. For the primary categorical analysis the prediction interval (0.98–7.79) includes the null, so a new EMS system could plausibly observe no early-versus-late difference at all even though the pooled estimate lies far from it. Only the non-shockable per-minute analysis has a prediction interval (0.94–0.98) that excludes the null, and even there the interval is narrow largely because only four studies contribute. Differences in EMS configuration, case mix and recorded time origin should therefore be expected to produce genuinely different timing gradients in different systems; the heterogeneity we report is better understood as real variation across systems than as noise to be averaged away.

### 4.3. Generalizability Across EMS Systems

Fourteen of the 25 included studies (56%), and most of the largest, are Japanese. This concentration reflects a genuine strength of the nationwide Fire and Disaster Management Agency Utstein registry, which captures every EMS-treated OHCA in the country with standardized, time-stamped documentation of the emergency call, EMS contact, defibrillation and drug administration [44]; few other systems can support per-minute timing analysis at this scale. It also means, however, that the quantitative core of this literature describes a single and distinctive prehospital system, and several features of that system bear directly on whether a timing gradient measured within it would translate elsewhere.

In Japan, epinephrine is administered by emergency life-saving technicians, the highest of the graded crew qualifications, acting under medical control, so whether a patient received epinephrine at all, and how quickly, partly reflects crew composition and the speed of medical direction in a way that it does not in systems operating under standing orders [11,33]; time-to-epinephrine in these cohorts therefore absorbs an authorization and vascular-access interval that such systems do not carry. Prehospital termination of resuscitation is also uncommon, and transport with CPR in progress is the norm rather than the exception, to the point that national registry data have been used to define when that decision should be taken—at about ten minutes after EMS-initiated CPR for shockable and eight minutes for non-shockable rhythms [45]. The patients who accumulate long times-to-epinephrine in Japan therefore include many in whom resuscitation would have been terminated on scene elsewhere, which changes who occupies the late-administration category and can change the magnitude of an observed gradient without any change in the underlying pharmacology. Working in the opposite direction, public-access defibrillation has been disseminated nationwide and its use in bystander-witnessed ventricular fibrillation rose from 1.1% in 2005 to 16.5% in 2013 [46]; an active bystander response of this kind shifts the case mix towards witnessed arrests with short no-flow intervals and compresses the distribution of times-to-epinephrine.

What these features have in common is that they act on the composition of the early and late groups rather than on the biology of epinephrine, and each can therefore alter the magnitude of a timing–outcome association without altering its direction. The pattern in our data is consistent with this. The direction of effect is reproduced outside Japan: US and US/Canadian cohorts contribute two of the four studies in the non-shockable per-minute estimate [10,31], and the Korean and Singaporean cohorts of Okada 2025 [18] show the same direction categorically. The largest and least plausible categorical magnitudes, by contrast, come from Japanese registry analyses [33,35]. We would therefore expect the qualitative conclusion, that avoidable delay is undesirable, to translate across EMS systems and the quantitative estimates, particularly the categorical odds ratios, not to. A system wishing to apply these results should first characterize its own time-to-epinephrine distribution, vascular-access practice and termination-of-resuscitation policy rather than assume that a gradient estimated in Japan describes its patients.

### 4.4. Limitations

Beyond the confounding and heterogeneity concerns discussed above, several additional limitations should be noted. First, although we searched three databases from inception without date restriction, the evidence base itself, dominated by registry-based observational cohorts with no timing-randomized trial, is the binding constraint on certainty, not the comprehensiveness of the search. Second, heterogeneous time reference points across studies (call-to-epinephrine, CPR-to-epinephrine, arrival-to-epinephrine, and defibrillation-to-epinephrine) are not clinically equivalent (for example, “10 min from call” and “10 min from EMS arrival” represent substantially different clinical scenarios), and this limits the precision and transferability of pooled estimates. Third, the geographical concentration in Japan (56%), with multiple studies drawing on overlapping FDMA registry data, limits generalizability; Section 4.3 sets out which features of that EMS system would be expected to affect the magnitude of a timing gradient elsewhere. Fourth, several subgroup analyses had very few studies (k = 2–3), and rhythm-stratified results from the primary categorical analysis should be considered exploratory. Fifth, although our primary analysis was restricted to direct adjusted comparisons, sensitivity analyses incorporating indirect comparisons and crude estimates yielded consistent results (OR range 2.55–3.78 across all sensitivity analyses, including restriction to a common time origin), suggesting a consistent direction but not resolving concerns about the precision of the effect magnitude. Sixth, GRADE certainty was “very low” for all eight assessed outcomes, reflecting the observational evidence base and serious risk of bias from residual confounding. Finally, the review was not registered in a prospective registry and no protocol was published in advance, which reduces protection against selective reporting and outcome switching. This is the limitation we can least mitigate, because it means that the boundary between prespecified and post hoc analysis rests on our own account rather than on an external record. As set out in Section 2.1, the review question, eligibility criteria, outcomes and rhythm stratification were fixed before extraction, and the dose–response and spline analyses were planned as exploratory secondary analyses, whereas the knot placement, the restriction of the primary categorical analysis to direct adjusted comparisons, the overlap rules and the E-value and time-origin sensitivity analyses were specified after the extracted data had been inspected and are post hoc. We report every analysis undertaken, including those that weakened the principal findings, namely the loss of the rhythm contrast under time-origin restriction, the prediction interval crossing the null in the primary categorical analysis, and the null randomized timing interaction; and we provide a fully reproducible deduplication, screening and study-selection procedure so that at least the selection steps can be checked independently of our account.

### 4.5. Implications and Future Directions

For **non-shockable rhythms**, the per-minute estimate shows between-study consistency (I^2^ = 0%) and aligns with current guideline recommendations for early epinephrine [2,3,5,6]. While the observational nature of the evidence precludes definitive causal inference, the consistency of findings across different EMS systems and time references suggests that efforts to minimize delays in epinephrine administration for non-shockable rhythms are reasonable.

For **shockable rhythms**, the evidence consistently favors early administration in direction but not in precise magnitude. However, the high heterogeneity, reliance on indirect comparisons and crude estimates in the subgroup analysis, and the priority of defibrillation in this population warrant caution against overinterpreting the magnitude of benefit. Kawakami 2024 [14] suggests that defibrillation-to-epinephrine intervals >6 min may be associated with worse outcomes, though this is a single-study finding requiring replication.

**What very low certainty means.** GRADE certainty was very low for all eight assessed outcomes, which means that the true effect is likely to be substantially different from these estimates and that no recommendation should rest on their magnitude. Three consequences follow. First, these results cannot support a numerical timing target: a recommendation of the form “administer epinephrine within X minutes” is not supportable from this evidence base, because the evidence cannot separate the effect of the clock from the effect of being the kind of patient who is reached early. Second, they can support the weaker and already established position that avoidable delay should be minimized, which is a statement about process quality and does not require a causal effect size to justify it. Third, for EMS systems, time-to-epinephrine is defensible as a process measure to be monitored and benchmarked, but it should not be adopted as an outcome-linked performance target: that would attach institutional consequences to a metric whose causal content is unknown and which is demonstrably confounded by patient trajectory. Guideline panels weighing this literature should note in particular that the only randomized timing evidence [38] is null and that the observational estimates are largest precisely where the design is weakest.

An **individual patient data meta-analysis** with harmonized time definitions would address the heterogeneous time reference problem, the single largest barrier to precise meta-analytic estimates in this literature. Future studies should use standardized time references, preferably EMS arrival-to-epinephrine as the most operationally relevant and least confounded interval, and employ time-dependent analytical methods to better address resuscitation time bias.

### 4.6. Key Messages

**What is established.** Epinephrine improves 30-day survival compared with placebo in adult OHCA, without a demonstrated improvement in neurological outcome [4]. Guidelines recommend it as early as feasible for non-shockable rhythms and after initial defibrillation attempts for shockable rhythms [2,3,5,6].

**What this review adds.** Across 25 observational studies, earlier epinephrine was associated with better outcomes in every analysis and every sensitivity analysis performed. The most internally consistent estimate is a per-minute gradient for non-shockable rhythm, approximately 4.2% lower survival odds for each minute of delay (OR 0.958, 95% CI 0.949–0.968; k = 4, I^2^ = 0%), which was stable across time origins, across sensitivity analyses and in leave-one-out analysis.

**What remains uncertain.** Whether any of these associations is causal. The magnitude of the categorical early-versus-late estimates, whose prediction interval (0.98–7.79) includes the null. Whether the timing gradient genuinely differs by rhythm, given that the apparent difference does not survive restriction to a common time origin. The shape of the time–response curve, and in particular whether any period exists during which delay is safe; we find no support for one.

**Main limitations.** All evidence is observational and subject to resuscitation time bias, which biases away from the null and cannot be removed by pooling; GRADE certainty is very low for all eight assessed outcomes; the time origins differ across studies and are not interchangeable; 56% of studies are Japanese and several share overlapping national registry data; and the review was not prospectively registered.

**Bottom line for practice.** Do not build a numerical timing target on this evidence. Continue to minimize avoidable delay to epinephrine, particularly in non-shockable rhythms, as a process-quality goal, and treat time-to-epinephrine as a monitored process measure rather than an outcome-linked performance target.

## 5. Conclusions

This systematic review found a consistent association between earlier epinephrine administration and improved outcomes in adult OHCA. The most internally consistent finding was for non-shockable rhythms, where each minute of delay was associated with approximately 4.2% lower survival odds (OR 0.958, I^2^ = 0%) across four studies, an estimate that was unchanged when restricted to studies sharing a single time origin. We describe this finding as internally consistent rather than reliable: agreement among four observational studies that share the same structural bias is evidence of consistency, not of validity, and the estimate remains a system-level description rather than a causal effect. Categorical analyses using direct adjusted comparisons favored early epinephrine for neurological outcome (OR 2.76, k = 9), with a consistent direction across all sensitivity analyses (OR range 2.55–3.78) but a prediction interval that includes the null. The apparent difference between rhythms did not persist once analyses were restricted to a common time origin. Exploratory time–response curves suggested declining outcomes beyond 15–20 min for both rhythms but should be regarded as hypothesis-generating given very high heterogeneity (I^2^ = 80–96%) arising from differing time reference points, and they do not identify a safe window. The certainty of evidence was very low for all outcomes, and the implausibly large magnitude of several estimates indicates substantial inflation by resuscitation time bias. These findings are consistent with current guideline recommendations to minimize avoidable delay, particularly in non-shockable rhythms, but they cannot define an optimal time or support a numerical timing target; that awaits trials that randomize timing and individual patient data meta-analyses with harmonized time definitions and analytical methods that adequately address time-dependent confounding.

## Figures and Tables

**Table 1:** Characteristics of Included Studies (N = 25).

Study	N	Rhythm	Time Reference	Outcomes	Adjustment	ROBINS-I	MA Role
Hayashi 2012, Japan [27]	3161	S/NS	Call → epi	CPC, Surv, ROSC	Yes (8 cov)	Serious	Narrative ¶
Nakahara 2012, Japan [28]	49,165	Overall	CPR → epi	CPC, Surv	Yes (9 cov)	Serious	Cat
Goto 2013, Japan [11]	209,577	S/NS	CPR → epi	CPC, Surv, ROSC	Yes (11 cov)	Serious	Cat †, DR, RCS
Koscik 2013, US [29]	686	S, PEA, Asys	Call → epi	ROSC, Surv	Partial	Critical	Narrative ^a^
Cantrell 2013, US [30]	660	Overall	Call → drug	ROSC	Yes (MV)	Serious	Narrative (ROSC)
Ewy 2015, US [31]	3469	S/NS	Dispatch → epi	CPC, Surv	Yes (HGLM)	Serious	PM
Hubble 2015, US [32]	1122	Overall	Call → vasopr	ROSC	Yes (MV)	Serious	Narrative (ROSC)
Fukuda 2016, Japan [33]	237,068	S/NS	Call → epi	CPC, Surv	PS matching	Serious	Cat †, DR, RCS
Tanaka 2016, Japan [26]	119,639	Overall	Call → epi	CPC, ROSC	Yes (12 cov)	Serious	Cat
Hubble 2017, US [34]	2100	Overall	Call → vasopr	CPC, Surv, ROSC	Yes (8 cov)	Serious	PM
Sagisaka 2017, Japan [35]	11,876	Overall	Call → epi	CPC, Surv	Yes (MV)	Serious	Cat
Funada 2018, Japan [12]	124,856	NS only	CPR → epi	CPC	Yes (7 cov)	Serious	Cat, DR, RCS
Hansen 2018, US/CA [10]	26,755	NS only	Arrival → epi	Surv, mRS	Yes (Utstein)	Serious	PM, Cat
Homma 2019, Japan [36]	9344	S/NS	Call → epi *	Surv, ROSC	Yes (GEE)	Serious	PM
Sigal 2019, US [37]	1826	S only	Call → epi	CPC, Surv	Yes (MV)	Serious	Narrative
Perkins 2020, UK [38]	4902	S/NS	Call → drug	ROSC, Surv, mRS	RCT	Moderate	Narrative
Okubo 2021, US/CA [13]	41,079	S/NS	Arrival → epi	Surv, mRS, ROSC	TD-PS	Moderate	Narrative †
Enzan 2022, Japan [16]	3050	NS (PEA)	Call → epi	CPC, Surv	Yes (8 cov)	Serious	PM, Cat
Fukuda 2021, Japan [9]	119,946	S/NS	Call → epi	CPC, Surv, ROSC	PS + MV	Serious	PM, Cat
Kawakami 2024, Japan [14]	20,905	S only	Defib → epi	CPC, Surv, ROSC	Yes (9 cov)	Serious	Cat, DR, RCS
Sakamoto 2025, Japan [20]	36,756	NS only	Contact → epi	CPC, Surv	TD-PS + GEE	Moderate	Narrative †
Otani 2026, Japan [17]	31,670	S/NS	Call → epi	CPC	Crude	Serious	Cat, DR, RCS
Izumida 2025, Japan [19]	184,731	S/NS	CPR → epi	CPC	Yes (MV)	Serious	Not pooled ‡
Okada 2025, KR/SG [18]	23,051	Overall	Arrival → epi	CPC, Surv	Yes (MV)	Serious	Cat
Gruebl 2025, Germany [21]	1090	S/PEA/Asys	Arrival → epi	Surv, ROSC	Bayesian	Serious	Narrative §

Registry, data years, and population details for the registry-based studies are provided in Table S4, and the timing categories used in the dose–response analyses are detailed in Table S3. Cat, categorical; CPC, favorable neurological outcome (CPC 1–2 or mRS ≤ 3); cov, covariates; DR, linear dose–response; MV, multivariable; NS, non-shockable; PM, per-minute; PS, propensity score; RCS, restricted cubic spline; S, shockable; Surv, survival; TD-PS, time-dependent propensity score. * Includes prehospital and in-hospital epinephrine. † Indirect comparison or TD-PS design (see Supplementary Methods). ‡ 95% CIs not reported. § Bayesian analysis only. ¶ Small n per rhythm subgroup. ^a^, No CPC data. → indicates the time origin (e.g., call → epi = call-to-epinephrine interval).

**Table 2:** Per-Minute Meta-Analysis (Pooled OR per 1-Minute Delay in Epinephrine).

Rhythm	Outcome	k	Total N	Pooled OR (95% CI)	95% PI	I^2^ (%)	*p* (Het)	Studies
**Non-shockable**	**Survival**	**4**	**40,449**	**0.958 (0.949–0.968)**	**0.94–0.98**	**0.0**	**0.89**	**Ewy 2015 [31], Hansen 2018 [10], Homma 2019 [36], Enzan 2022 [16]**
Non-shockable	Neuro (CPC/mRS)	3	31,823	0.953 (0.929–0.978)	0.81–1.13	0.0	0.75	Ewy 2015 [31], Hansen 2018 [10], Enzan 2022 [16]
Shockable	Survival	2	2169	0.980 (0.904–1.061)	—	93.7	<0.001	Ewy 2015 [31], Homma 2019 [36]
Shockable	Neuro (CPC)	1	1451	0.98 (0.92–1.04) *	—	—	—	Ewy 2015 [31]
Overall	CPC 1–2	2	122,046	0.910 (0.900–0.919)	—	0.0	—	Fukuda 2021 [9], Hubble 2017 [34]

k, number of studies; OR, odds ratio; CI, confidence interval; I^2^, measure of statistical heterogeneity; *p* (Het), *p*-value for heterogeneity. 95% PI, 95% prediction interval (Higgins–Thompson–Spiegelhalter), the range within which the true effect of a future study is expected to fall; — denotes not estimable (fewer than 3 studies). Prediction intervals based on few studies (k ≤ 4) are very wide and should be interpreted with caution. * Single study; not pooled. Rows set in bold indicate the per-minute non-shockable survival gradient, which is precise, consistent between studies and unchanged by restriction to a common time origin. Abbreviation definitions provided in Table 2 apply to the subsequent tables.

**Table 3:** Primary Analysis: Direct Adjusted Comparisons Only.

Analysis	k	Pooled OR (95% CI)	95% PI	I^2^ (%)	Studies
**All rhythms combined**	**9**	**2.76 (2.06–3.71)**	**0.98–7.79**	**88.5**	**Enzan 2022 (NS) [16], Fukuda 2021 (S + NS) [9], Kawakami 2024 (S) [14], Funada 2018 (NS) [12], Nakahara 2012 (O) [28], Tanaka 2016 (O) [26], Sagisaka 2017 (O) [35], Okada 2025 Korea (O) [18]**
— Non-shockable	3	2.45 (2.04–2.94)	0.75–8.03	0.0	Enzan 2022 [16], Fukuda 2021 [9], Funada 2018 [12]
— Shockable	2	3.81 (2.52–5.77)	—	87.8	Fukuda 2021 [9], Kawakami 2024 [14]
— Overall	4	2.43 (1.35–4.38)	0.15–39.69	90.2	Nakahara 2012 [28], Tanaka 2016 [26], Sagisaka 2017 [35], Okada 2025 Korea [18]

Prediction intervals based on few studies (k ≤ 4) are very wide and should be interpreted with caution. Between-subgroup test (S vs. NS): *p* = 0.053 (not significant); three-group test (S/NS/Overall): *p* = 0.15. Rows set in bold indicate the primary categorical neurological-outcome analysis restricted to direct adjusted comparisons. S, shockable rhythm; NS, non-shockable rhythm; O, overall.

**Table 4:** Sensitivity Analysis: All Comparisons (Including Indirect and Crude).

Rhythm Subgroup	k	Pooled OR (95% CI)	95% PI	I^2^ (%)	Direct Studies	Indirect Studies †
All rhythms combined	15	3.19 (2.50–4.06)	1.22–8.31	86.3	Enzan 2022 [16], Fukuda 2021 (S + NS) [9], Otani 2026 (S + NS) ^c^ [17], Kawakami 2024 [14], Funada 2018 [12], Nakahara 2012 [28], Tanaka 2016 [26], Sagisaka 2017 [35], Okada 2025 Korea [18]	Goto 2013 (S + NS) [11], Fukuda 2016 (S + NS) [33]
Shockable	5	4.31 (3.29–5.65)	1.72–10.81	77.9	Fukuda 2021 [9], Otani 2026 ^c^ [17], Kawakami 2024 [14]	Goto 2013 [11], Fukuda 2016 [33]
Non-shockable	6	2.80 (2.21–3.56)	1.54–5.10	40.6	Enzan 2022 [16], Fukuda 2021 [9], Otani 2026 ^c^ [17], Funada 2018 [12]	Goto 2013 [11], Fukuda 2016 [33]
Overall (not stratified)	4	2.43 (1.35–4.38)	0.15–39.69	90.2	Nakahara 2012 [28], Tanaka 2016 [26], Sagisaka 2017 [35], Okada 2025 Korea [18]	—

Prediction intervals based on few studies (k ≤ 4) are very wide and should be interpreted with caution. † Indirect comparisons: ORs derived from early-category and late-category ORs relative to shared no-epinephrine reference group. ^c^ Crude (unadjusted) OR. Between-subgroup test: shockable vs. non-shockable χ^2^ = 5.45, df = 1, *p* = 0.020; three-group (S/NS/Overall) χ^2^ = 6.57, df = 2, *p* = 0.038. The rhythm difference is statistically significant in this comprehensive analysis but not in the primary direct adjusted analysis (S-vs-NS *p* = 0.053; three-group *p* = 0.15), indicating that it emerges only when lower-quality indirect and unadjusted comparisons are added. It is likewise absent when the comprehensive analysis is restricted to estimates sharing a common time origin (*p* = 0.46; Table S5). S, shockable rhythm; NS, non-shockable rhythm.

**Table 5:** Categorical Survival Meta-Analysis.

Analysis	k	Pooled OR (95% CI)	95% PI	I^2^ (%)	Studies
Survival (mixed rhythm)	6	2.08 (1.48–2.93)	0.61–7.05	92.4	Hansen 2018 (NS) [10], Nakahara 2012 [28], Okada 2025 Korea [18], Okada 2025 Singapore [18], Goto 2013 † (NS) [11], Sagisaka 2017 (O) [35]

† Indirect comparison: OR derived from early-category and late-category ORs relative to a shared no-epinephrine reference group. NS, non-shockable rhythm; O, overall.

**Table 6:** Linear Time–Response Meta-Analysis (Greenland–Longnecker, CPC 1–2).

Rhythm	k	Pooled OR per Min (95% CI)	I^2^ (%)	Studies
Shockable	4	0.941 (0.919–0.963)	96.0	Goto 2013 [11], Fukuda 2016 [33], Kawakami 2024 [14], Otani 2026 [17]
Non-shockable	4	0.971 (0.957–0.985)	83.1	Goto 2013 [11], Fukuda 2016 [33], Funada 2018 [12], Otani 2026 [17]

Between-group test (S vs. NS linear slope): *p* = 0.067.

**Table 7:** Pooled Restricted Cubic Spline Time–Response (Exploratory; CPC 1–2, Reference: 10 min).

Rhythm	k	Time (min)	OR vs. 10 min (95% CI)	I^2^ (%)
Shockable	4	5	1.44 (1.09–1.90)	94.9
		15	0.71 (0.59–0.85)	
		20	0.52 (0.42–0.64)	
		25	0.39 (0.34–0.44)	
		30	0.29 (0.24–0.35)	
Non-shockable	4	5	1.06 (0.88–1.28)	79.6
		15	0.89 (0.80–1.00)	
		20	0.73 (0.64–0.82)	
		25	0.55 (0.47–0.64)	
		30	0.40 (0.30–0.54)	

Studies—Shockable: Goto 2013 [11], Fukuda 2016 [33], Kawakami 2024 [14], Otani 2026 [17]; Non-shockable: Goto 2013 [11], Fukuda 2016 [33], Funada 2018 [12], Otani 2026 [17].

## Data Availability

The extracted data and analysis code are available from the corresponding author upon reasonable request.

## Supplementary Materials

The following supporting information can be downloaded at: https://www.mdpi.com/article/10.3390/medicina62081495/s1, Figure S1: Sensitivity analysis for RCS knot placement, Figure S2: Funnel plot for categorical CPC 1–2 meta-analysis (k=15). Direct comparisons shown as blue circles; indirect comparisons (†) shown as red triangles. Study labels include rhythm abbreviation (S = shockable, NS = non-shockable, O = overall). The four indirect estimates (Goto 2013 [11] and Fukuda 2016 [33]) lie outside the pseudo-95% confidence region, consistent with the known upward bias of indirect OR ratios rather than selective publication, Figure S3: Funnel plot for per-minute non-shockable survival meta-analysis (k = 4). With only four studies, this plot is presented for visual inspection only; formal tests of funnel-plot asymmetry are underpowered and unreliable with fewer than 10 studies, so publication bias could not be assessed [47], Figure S4: Leave-one-out sensitivity analysis for per-minute non-shockable survival, Figure S5: Influence diagnostics for per-minute non-shockable survival, Figure S6: Forest plot: Per-minute OR for non-shockable survival (k = 4), Figure S7: Combined time–response comparison: shockable vs. non-shockable rhythm restricted cubic spline curves overlaid; Table S1: PRISMA 2020 Checklist, Table S2: Search Strategies, Table S3: Time-Response Data Extraction. Detailed data for studies contributing to the two-stage time-response meta-analysis (linear and RCS). For each study, timing categories, midpoint assignments, reference category, effect estimates, cell counts (where available), and time reference point are provided, Table S4: Registry Overlap Assessment, Table S5: Sensitivity Analysis Restricted to a Common Time Origin. Included studies did not share a common origin for “time to epinephrine”. This table re-runs the principal analyses after restricting them to studies that share a single origin, and tests the time origin as a subgroup variable. All models are inverse-variance random-effects (REML) on the log-OR scale, identical in specification to the corresponding main-text analysis; prediction intervals use the Higgins–Thompson–Spiegelhalter method. R 4.5.2, meta 8.2.1., Table S6: E-value Sensitivity Analysis for Unmeasured Confounding, Table S7: Sensitivity Analyses for Categorical CPC 1–2 Meta-Analysis, Table S8: Leave-One-Out Sensitivity Analysis (Per-Minute Non-Shockable Survival, k = 4), Table S9: ROBINS-I Risk of Bias Assessment, Table S10: GRADE Summary of Findings.
