# Supplementary material for: Association Between Epinephrine Administration Timing and Outcomes in Adult Out-of-Hospital Cardiac Arrest: A Systematic Review and Dose–Response Meta-Analysis
Source: Medicina (Kaunas). 2026 Aug 3;62(8):1495. doi: 10.3390/medicina62081495 (PMC13515038; doi:10.3390/medicina62081495)
Supplement: Supplementary file 1 [file medicina-62-01495-s001.zip › medicina-4449421-supplementary.pdf]

Supplementary Materials

Association Between Epinephrine Administration Timing and Outcomes in Adult Out-of-Hospital Cardiac Arrest: A Systematic Review and Dose-Response Meta-Analysis

Chiwon Ahn, Jae Hwan Kim, So June Hwang, Young Taeck Oh

Supplementary Methods

Search deduplication and screening. The 5,953 records retrieved from PubMed (n=1,135), Embase (n=4,527), and the Cochrane Central Register of Controlled Trials (n=291) were deduplicated by exact matching of normalized titles (lowercased, punctuation and whitespace removed, Unicode folded to ASCII) and digital object identifiers, with records sharing a title or DOI merged into a single record (union–find clustering). This removed 1,555 duplicates, leaving 4,398 unique records. A reproducible relevance screen was then applied to titles: a record was retained if its title contained a cardiac-arrest term (cardiac arrest, heart arrest, OHCA, cardiopulmonary resuscitation, CPR, ROSC, return of spontaneous circulation, or out-of-hospital) AND an intervention term (epinephrine, adrenaline, adrenalin, or vasopressor), AND did not contain an exclusion term (pediatric, paediatric, child(ren), infant, neonatal, newborn, in-hospital/IHCA, animal/porcine/swine/rat/mouse/mice/canine/pig/dog, sepsis/septic, trauma, burn, or h(a)emorrhagic); the 25 included studies were verified to all pass this screen. This yielded 227 reports assessed for eligibility, of which 202 were excluded with reasons (Figure 1) and 25 met all eligibility criteria. This automated screen is fully reproducible from the search exports, which are available from the corresponding author upon reasonable request; titles lacking explicit timing terms (e.g., studies whose timing analysis was reported only in the abstract or full text) were retained at this stage and resolved at full-text eligibility assessment.

Indirect comparison calculation. For studies reporting timing-category outcomes relative to a shared no-epinephrine reference group, indirect early-versus-late odds ratios were calculated as: log(OR_indirect) = log(OR_early/ref) − log(OR_late/ref), with variance estimated by simple summation: Var(log OR_indirect) = Var(log OR_early/ref) + Var(log OR_late/ref) (Bucher method assuming independence). Specifically, Goto 2013 [11] compared the earliest (≤9 min) versus latest (≥20 min) categories, and Fukuda 2016 [33] compared the earliest (0–15 min) versus latest (≥31 min) categories, each relative to a shared no-epinephrine reference. Because the early and late categories share a common reference, their log-OR estimates are positively correlated; assuming independence therefore overestimates the variance (yielding conservatively wider CIs). These indirect comparisons are identified with a dagger (†) in forest plots.

Time-response model details. Two-stage dose-response meta-analysis methods were applied to time-to-epinephrine as the continuous exposure variable (the term "dose-response" is standard in the dosresmeta methodology but here refers to time-to-treatment). In stage 1, study-specific RCS coefficients were estimated using generalized least squares, with within-study correlation modeled using the Greenland-Longnecker method for studies providing reference-category cell counts (Goto 2013, Fukuda 2016, Funada 2018), or assuming independence for studies reporting only adjusted estimates (Kawakami 2024, Otani 2025). In stage 2, coefficients were pooled using multivariate random-effects meta-analysis (REML). An approximate I^2 was derived as max(0,

100 × (Q − df) / Q). Given that contributing studies used different time reference points (CPR-to-epinephrine, call-to-epinephrine, defibrillation-to-epinephrine), the pooled curves represent an exploratory synthesis of the overall time-response shape rather than precise estimates at specific clock times.

Heterogeneity interpretation. I^2 values were interpreted as approximate guides: 0–40% unimportant, 30–60% moderate, 50–90% substantial, 75–100% considerable [40].

Publication bias. Funnel plots were constructed for the categorical CPC 1–2 analysis (Figure S2) and the per-minute non-shockable survival analysis (Figure S3). Because most analyses in this review included fewer than 10 studies, formal statistical tests for funnel-plot asymmetry (e.g., Egger's regression) were not performed, in keeping with Cochrane guidance that such tests are underpowered and unreliable below this threshold [47]. The funnel plots are therefore provided for visual inspection only and should not be interpreted as confirming or excluding publication bias; consequently, publication bias could not be formally assessed for any outcome and was not used as a GRADE downgrading criterion. A further reason not to apply Egger's test to the categorical analysis is that it combines heterogeneous effect measures (adjusted ORs, propensity-score-matched RRs, and indirect OR ratios), violating the assumption of a common underlying effect [47].

Table S1. PRISMA 2020 Checklist

Manuscript: Association Between Epinephrine Administration Timing and Outcomes in Adult Out-of-Hospital Cardiac Arrest: A Systematic Review and Dose-Response Meta-Analysis

Locations refer to sections of the manuscript and to the Supplementary Materials.

Section / Topic	#	Checklist item	Location reported
**TITLE**			
Title	1	Identify the report as a systematic review.	Title ("A Systematic Review and Dose-Response Meta-Analysis")
**ABSTRACT**			
Abstract	2	See the PRISMA 2020 for Abstracts checklist.	Abstract (Background and Objectives / Materials and Methods / Results / Conclusions)
**INTRODUCTION**			
Rationale	3	Describe the rationale for the review in the context of existing knowledge.	1. Introduction (paragraphs 1–2)
Objectives	4	Provide an explicit statement of the objective(s) or question(s) the review addresses.	1. Introduction (final paragraph; aims 1–4)
**METHODS**			
Eligibility criteria	5	Specify the inclusion and exclusion criteria for the review and how studies were grouped for the syntheses.	2.2 Eligibility Criteria; 2.5 (Overlap management)
Information	6	Specify all databases, registers, websites, etc.	2.3 Search Strategy (PubMed, Embase,

Section / Topic	#	Checklist item	Location reported
sources		searched, and the date each was last searched.	CENTRAL; inception–15 March 2026); Table S2
Search strategy	7	Present the full search strategies for all databases, including any filters and limits used.	2.3 Search Strategy; Table S2 (full strategies)
Selection process	8	Specify the methods used to decide whether a study met inclusion criteria, including number of reviewers, independence, and any automation tools.	2.4 Study Selection and Data Extraction; Supplementary Methods ("Search deduplication and screening" — reproducible automated screen)
Data collection process	9	Specify the methods used to collect data from reports, number of reviewers, independence, and any automation tools.	2.4 Study Selection and Data Extraction
Data items	10a	List and define all outcomes for which data were sought; whether all compatible results were sought.	2.2 Eligibility Criteria; 2.5 (Outcome definitions)
Data items	10b	List and define all other variables for which data were sought; assumptions about missing/unclear information.	2.4 Data Extraction; Table 1; Table S3
Study risk of bias assessment	11	Specify methods to assess risk of bias, the tool(s) used, number of reviewers, independence.	2.4 (ROBINS-I); Table S9
Effect measures	12	Specify for each outcome the effect measure(s) used.	2.5 Statistical Analysis (odds ratio; per-minute OR)
Synthesis methods	13a	Describe processes to decide which studies were eligible for each synthesis.	2.5 Statistical Analysis; 2.5 (Overlap management)
Synthesis methods	13b	Describe methods to prepare data for synthesis (missing statistics, data conversions).	2.5 (log-OR scale; OR–RR equivalence; Bucher indirect comparison); Supplementary Methods
Synthesis methods	13c	Describe methods to tabulate or visually display results of individual studies and syntheses.	2.5; Table 2; Figures 2–3
Synthesis methods	13d	Describe methods used to synthesize results, the model(s), heterogeneity assessment, and software.	2.5 (inverse-variance random-effects REML; two-stage dose-response/RCS; I^2 and Cochran's Q; R 4.5.2 — meta, metafor, mvmeta, dosresmeta)
Synthesis methods	13e	Describe methods used to explore possible causes of heterogeneity (e.g., subgroup analysis).	2.5 (rhythm-stratified subgroups); 3.3
Synthesis methods	13f	Describe sensitivity analyses conducted to assess robustness.	2.5 (Subgroup and sensitivity analyses); Table S7; Table S4
Reporting bias assessment	14	Describe methods to assess risk of bias due to missing results (reporting biases).	2.5; Supplementary Methods (Publication bias); Figures S2–S3
Certainty assessment	15	Describe methods to assess certainty in the body of evidence.	2.5 (GRADE); Table S10
**RESULTS**			
Study selection	16a	Describe results of search and selection, ideally using a flow diagram.	3.1 Study Selection and Characteristics; Figure 1 (PRISMA flow)
Study selection	16b	Cite studies that appeared to meet inclusion criteria but were excluded, and explain why.	3.1 (e.g., Matsuyama 2022 — epinephrine use vs non-use); Figure 1; 3.6 Narrative

Section / Topic	#	Checklist item	Location reported
			Synthesis
Study characteristics	17	Cite each included study and present its characteristics.	3.1; Table 1; Table S4
Risk of bias in studies	18	Present risk-of-bias assessments for each included study.	3.5 Risk of Bias and Certainty of Evidence; Table S9
Results of individual studies	19	For all outcomes, present for each study an effect estimate and its precision, ideally using tables/plots.	Figures 2–3; Table 2; Table S3 (time-response data extraction)
Results of syntheses	20a	For each synthesis, summarise characteristics and risk of bias among contributing studies.	3.2–3.4; 3.5
Results of syntheses	20b	Present results of all statistical syntheses; summary estimate, precision, heterogeneity; direction of effect.	3.2–3.4; Table 2; Figures 2–3
Results of syntheses	20c	Present results of all investigations of possible causes of heterogeneity.	3.3 (rhythm subgroups; between-subgroup tests); 3.4
Results of syntheses	20d	Present results of all sensitivity analyses.	3.3; Table S7; Table S4 (registry-overlap sensitivity)
Reporting biases	21	Present assessments of risk of bias due to missing results for each synthesis assessed.	Supplementary Methods (Publication bias); Figures S2–S3
Certainty of evidence	22	Present assessments of certainty in the body of evidence for each outcome.	3.5; Table S10 (GRADE summary of findings)
**DISCUSSION**			
Discussion	23a	Provide a general interpretation of the results in the context of other evidence.	4.1 Principal Findings and What This Review Adds; 4.2 Confounding and Causal Inference
Discussion	23b	Discuss any limitations of the evidence included in the review.	4.2; 4.3 Limitations
Discussion	23c	Discuss any limitations of the review processes used.	4.3 Limitations (non-registration; title-based reproducible screening; heterogeneous time references)
Discussion	23d	Discuss implications of the results for practice, policy, and future research.	4.4 Implications and Future Directions; 5. Conclusions
**OTHER INFORMATION**			
Registration and protocol	24a	Provide registration information, or state that the review was not registered.	2.1 Study Design and Registration — **the review was not registered**
Registration and protocol	24b	Indicate where the protocol can be accessed, or state that a protocol was not prepared.	2.1 — **no protocol was published or registered in advance**
Registration and protocol	24c	Describe and explain any amendments to registration/protocol information.	Not applicable (no registration or protocol)
Support	25	Describe sources of financial/non-financial support and the role of funders.	Funding (back matter; KHIDI grant RS-2024-00335936; funder had no role)
Competing interests	26	Declare any competing interests of review authors.	Conflicts of Interest (back matter — none declared)
Availability of data, code and other materials	27	Report which materials are publicly available and where (data, analytic code, etc.).	Data Availability Statement; Supplementary Materials; extracted data and analysis/screening code available

Section / Topic	#	Checklist item	Location reported
			from the corresponding author

From: Page MJ, McKenzie JE, Bossuyt PM, et al. The PRISMA 2020 statement: an updated guideline for reporting systematic reviews. *BMJ* 2021;372:n71. doi:10.1136/bmj.n71. For more information, visit http://www.prisma-statement.org/.

Table S2. Search Strategies

Cochrane Library (Title/Abstract/Keyword)

Block	Search Terms
#1 Population	cardiac arrest OR heart arrest OR cardiopulmonary resuscitation OR CPR OR OHCA OR ventricular fibrillation OR pulseless electrical activity OR PEA OR asystole
#2 Intervention	epinephrine OR adrenaline OR adrenalin OR vasopressor
#3 Exposure	timing OR early OR delay OR interval OR rapid OR minutes
#4 Outcome	survival OR mortality OR neurological outcome OR neurologic outcome OR cerebral performance OR CPC OR return of spontaneous circulation OR ROSC OR favourable outcome OR favorable outcome
#5 Final	#1 AND #2 AND #3 AND #4 — **291 records** (database inception through 2026/03/15; no date or language restriction)

PubMed

Block	Search Terms
#1 Population	(“Heart Arrest”[MeSH] OR “Cardiopulmonary Resuscitation”[MeSH] OR “Out-of-Hospital Cardiac Arrest”[MeSH] OR cardiac arrest[tiab] OR heart arrest[tiab] OR cardiopulmonary resuscitation[tiab] OR CPR[tiab] OR OHCA[tiab] OR ventricular fibrillation[tiab] OR pulseless electrical activity[tiab] OR PEA[tiab] OR asystole[tiab])
#2 Intervention	(“Epinephrine”[MeSH] OR epinephrine[tiab] OR adrenaline[tiab] OR adrenalin[tiab] OR vasopressor[tiab])
#3 Exposure	(“Time-to-Treatment”[MeSH] OR timing[tiab] OR time to[tiab] OR early[tiab] OR delay[tiab] OR interval[tiab] OR rapid[tiab] OR minutes[tiab] OR administration time[tiab])
#4 Outcome	(survival[tiab] OR mortality[tiab] OR neurological outcome[tiab] OR neurologic outcome[tiab] OR cerebral performance[tiab] OR CPC[tiab] OR return of spontaneous circulation[tiab] OR ROSC[tiab] OR favourable outcome[tiab] OR favorable outcome[tiab])
#5 Final	#1 AND #2 AND #3 AND #4 — **1,135 records** (database inception through 2026/03/15; no date or language restriction)

Embase (embase.com)

Block	Search Terms
#1 Population	‘heart arrest’/exp OR ‘cardiopulmonary resuscitation’/exp OR (cardiac NEAR/1 arrest OR heart NEAR/1 arrest OR cardiopulmonary NEAR/1 resuscitation OR cpr OR ohca OR ventricular NEAR/1 fibrillation OR pulseless NEAR/2 electrical NEAR/2 activity OR pea OR asystole):ti,ab,kw
#2 Intervention	‘adrenaline’/exp OR (epinephrine OR adrenaline OR adrenalin OR vasopressor):ti,ab,kw
#3 Exposure	‘time to treatment’/exp OR (timing OR early OR delay OR interval OR

Block	Search Terms
	rapid OR minutes OR ‘administration time’):ti,ab,kw
#4 Outcome	(survival OR mortality OR ‘neurological outcome’ OR ‘neurologic outcome’ OR ‘cerebral performance’ OR cpc OR ‘return of spontaneous circulation’ OR rosc OR ‘favourable outcome’ OR ‘favorable outcome’):ti,ab,kw
#5 Final	#1 AND #2 AND #3 AND #4 — **4,527 records** (database inception through 2026/03/15; no date or language restriction)

Note. No search-level exclusion filters were applied. Pediatric, in-hospital, and animal records, and records addressing route of administration or dosing interval rather than time-to-first-dose, were removed during title/abstract and full-text screening. Search-level “NOT child/children” filters were deliberately avoided after pilot testing showed they discarded eligible mixed adult-and-pediatric cohorts (e.g., a large non-shockable OHCA cohort spanning children and adults). Combined across the three databases, 5,953 records were retrieved (PubMed 1,135; Embase 4,527; CENTRAL 291).

Table S3. Time-Response Data Extraction

Detailed data for studies contributing to the two-stage time-response meta-analysis (linear and RCS). For each study, timing categories, midpoint assignments, reference category, effect estimates, cell counts (where available), and time reference point are provided.

Goto 2013 (Shockable, N=15,492; Non-shockable, N=194,085; Time ref: CPR start → epi; Ref: no epi)

Rhythm	Category	Midpoint (min)	CPC 1–2 aOR	95% CI
Shockable	≤9 min	5	0.71	0.54–0.92
Shockable	10–19 min	15	0.34	0.28–0.42
Shockable	≥20 min	25	0.21	0.14–0.31
Non-shockable	≤9 min	5	0.95	0.62–1.37
Non-shockable	10–19 min	15	0.63	0.48–0.80
Non-shockable	≥20 min	25	0.49	0.32–0.71

Fukuda 2016 (Shockable, N=16,616; Non-shockable, N=220,452; Time ref: EMS call → epi; Ref: no epi)

Rhythm	Category	Midpoint (min)	CPC 1–2 OR	95% CI
Shockable	0–15	8	1.95	1.37–2.72
Shockable	16–20	18	0.90	0.66–1.20
Shockable	21–25	23	0.41	0.28–0.58
Shockable	26–30	28	0.21	0.11–0.36
Shockable	≥31	33	0.26	0.15–0.42
Non-shockable	0–15	8	1.72	0.98–2.80
Non-shockable	16–20	18	1.38	0.95–1.95
Non-shockable	21–25	23	1.03	0.71–1.43

Rhythm	Category	Midpoint (min)	CPC 1–2 OR	95% CI
Non-shockable	26–30	28	0.41	0.22–0.69
Non-shockable	≥31	33	0.30	0.15–0.52

Funada 2018 (Non-shockable only, N=124,856; Time ref: CPR start → epi; Ref: epi ≥20 min)

Category	Midpoint (min)	CPC 1–2 aOR	95% CI
≤10 min	5	2.66	1.63–4.55
11–19 min	15	2.00	1.24–3.40
≥20 min (ref)	25	1.00	—

Kawakami 2024 (Shockable only, N=20,905; Time ref: Defibrillation → epi; Ref: 4–6 min)

Category	Midpoint (min)	CPC 1–2 aOR	95% CI
<2	1	0.70	0.37–1.33
2–4	3	1.14	0.87–1.48
4–6 (ref)	5	1.00	—
6–8	7	0.63	0.53–0.76
8–10	9	0.61	0.51–0.73
10–12	11	0.42	0.34–0.51
12–14	13	0.41	0.33–0.50
14–16	15	0.30	0.24–0.38
16–18	17	0.26	0.20–0.35
≥18	20	0.21	0.16–0.26

Otani 2025 (Shockable, N=5,859; Non-shockable, N=25,811; Time ref: EMS call → epi; Crude OR)

Rhythm	Category	Midpoint (min)	CPC 1–2 events/N	Crude OR
Shockable	EAG 0–19	10	398/2,275	1.00 (ref)
Shockable	IAG 20–26	23	147/2,137	0.35
Shockable	LAG 27–56	42	58/1,447	0.19
Non-shockable	EAG 0–19	10	123/8,321	1.00 (ref)
Non-shockable	IAG 20–26	23	81/9,461	0.57
Non-shockable	LAG 27–56	42	33/8,029	0.27

Table S4. Registry Overlap Assessment

A. Japanese Study Registry Map

Study	Registry	Data Years	Population	Rhythm
Hayashi 2012	Osaka Utstein (regional)	2007–2009	Witnessed, cardiac	S + NS
Nakahara 2012	FDMA (national)	2007–2008	Witnessed, early ROSC excluded	Overall
Goto 2013	FDMA	2009–2010	All OHCA	S + NS
Fukuda 2016	FDMA	2011–2012	All OHCA	S + NS

Study	Registry	Data Years	Population	Rhythm
Tanaka 2016	FDMA	2008–2012	Witnessed, cardiac	Overall
Sagisaka 2017	FDMA	2010–2012	Witnessed, cardiogenic	Overall
Funada 2018	FDMA	2011–2014	Witnessed, bystander CPR	NS only
Homma 2019	SOS-KANTO (regional)	2012–2013	All OHCA	S + NS
Enzan 2022	JAAM-OHCA + Utstein	2014–2016	PEA only	NS only
Fukuda 2021	FDMA	2011–2017	All OHCA	S + NS
Kawakami 2024	FDMA	2011–2020	Witnessed, cardiac	S only
Sakamoto 2025	JAAM-OHCA + Utstein	2014–2020	Epi recipients	NS only
Otani 2025	FDMA (Utstein)	2015–2019	Bystander CPR	S + NS
Izumida 2025	FDMA (Utstein)	2006–2021	All OHCA	S + NS

FDMA = Fire and Disaster Management Agency nationwide Utstein registry; JAAM-OHCA = Japanese Association for Acute Medicine OHCA registry (multi-center, overlaps partially with FDMA Utstein data); SOS-KANTO = Survey of Survivors after Out-of-Hospital Cardiac Arrest in Kanto area (regional prospective registry).

B. FDMA Registry Overlap Timeline

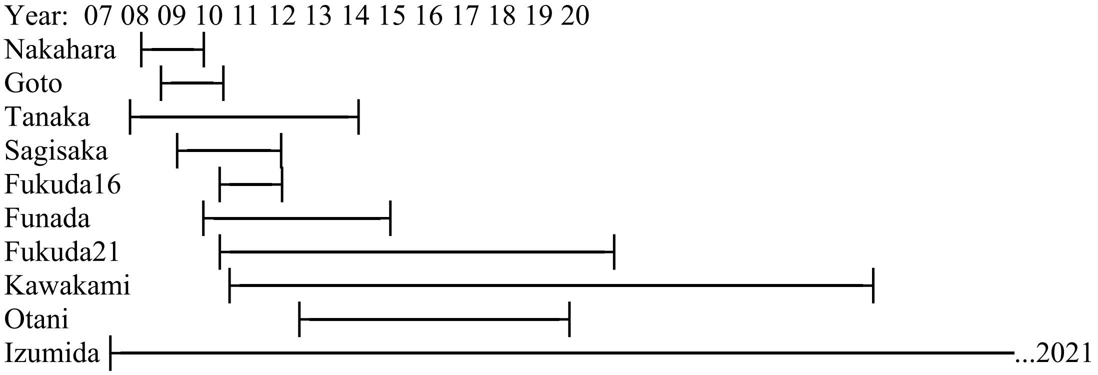

C. Overlap Management by Analysis

Per-minute meta-analysis (NS survival, k=4): Ewy 2015 (US), Hansen 2018 (US/CA), Homma 2019 (SOS-KANTO), Enzan 2022 (JAAM-OHCA). No FDMA national registry studies included; no overlap concern. Homma 2019 uses the regional SOS-KANTO registry and Enzan 2022 uses the JAAM-OHCA registry, which are independent data sources.

Primary categorical analysis (direct adjusted, k=9):

Estimate	Registry	Years	Potential Overlap
Nakahara 2012 (O)	FDMA	2007–2008	Tanaka 2016 (2008 overlap)
Tanaka 2016 (O)	FDMA	2008–2012	Nakahara (2008), Sagisaka 2017 (2010–2012), Fukuda 2021 (2011–2012)
Sagisaka 2017 (O)	FDMA	2010–2012	Tanaka 2016 (2010–2012), Fukuda 2021 (2011–2012)

Estimate	Registry	Years	Potential Overlap
Funada 2018 (NS)	FDMA	2011–2014	Fukuda 2021 NS (2011–2014)
Fukuda 2021 (S)	FDMA	2011–2017	Kawakami 2024 S (2011–2017)
Fukuda 2021 (NS)	FDMA	2011–2017	Funada 2018 NS (2011–2014)
Kawakami 2024 (S)	FDMA	2011–2020	Fukuda 2021 S (2011–2017)
Enzan 2022 (NS)	JAAM-OHCA	2014–2016	Independent registry
Okada 2025 (O)	KOHCAR/PAROS	2018–2022	No overlap (Korea/Singapore)

Key overlaps allowed in primary analysis: 1. **Fukuda 2021 S + Kawakami 2024 S**: Both use FDMA with overlapping years (2011–2017). However, Kawakami uniquely analyzes defibrillation-to-epinephrine timing, while Fukuda 2021 uses call-to-epinephrine, providing complementary evidence on different time references. These contribute to the same shockable subgroup. 2. **Fukuda 2021 NS + Funada 2018 NS**: Overlapping FDMA data (2011–2014). Funada 2018 restricts to bystander-witnessed CPR; Fukuda 2021 includes all OHCA. Partial population overlap is present. 3. **Nakahara 2012 + Tanaka 2016**: 1-year overlap (2008), minimal given different study periods and inclusion criteria. 4. **Sagisaka 2017 + Tanaka 2016**: Both use FDMA mixed-rhythm data with substantial period overlap (2010–2012). Both contribute to the overall subgroup. They differ in exposure definition (Sagisaka analyzes call-to-epinephrine tertiles in witnessed cardiogenic arrest with repeated-dose adjustment; Tanaka uses a 19-minute call-to-epinephrine threshold), but the population overlap is material and was addressed by dedicated leave-one-out analyses.

Mitigation: A sensitivity analysis using non-overlapping registries only (k=8; selecting one study per FDMA period) yielded OR 3.12 (95% CI 2.20–4.44; I^2=91.9%), consistent with the comprehensive analysis. In addition, leave-one-out analyses removing each of the two overlapping mixed-rhythm studies bracketed the comprehensive estimate: excluding Sagisaka 2017, OR 3.05 (2.40–3.89); excluding Tanaka 2016, OR 3.26 (2.51–4.23). The pooled estimate is therefore robust to the FDMA overlap.

Time-response RCS analysis (k=4 per rhythm):

Shockable (k=4)	Registry	Years	Overlap
Goto 2013	FDMA	2009–2010	None with others in this analysis
Fukuda 2016	FDMA	2011–2012	Kawakami (2011–2012)
Kawakami 2024	FDMA	2011–2020	Fukuda 2016 (2011–2012), Otani (2015–2019)
Otani 2025	FDMA	2015–2019	Kawakami (2015–2019)

Non-shockable (k=4)	Registry	Years	Overlap
Goto 2013	FDMA	2009–2010	None with others in this analysis
Fukuda 2016	FDMA	2011–2012	Funada (2011–2012)
Funada 2018	FDMA	2011–2014	Fukuda 2016 (2011–2012)

Non-shockable (k=4)	Registry	Years	Overlap
Otani 2025	FDMA	2015–2019	None with others for NS

Overlap is present in the time-response analysis and contributes to the very high heterogeneity (I^2=80–95%). These results are accordingly labeled as exploratory throughout the manuscript.

Table S5. Sensitivity Analysis Restricted to a Common Time Origin

Included studies did not share a common origin for "time to epinephrine". This table re-runs the principal analyses after restricting them to studies that share a single origin, and tests the time origin as a subgroup variable. All models are inverse-variance random-effects (REML) on the log-OR scale, identical in specification to the corresponding main-text analysis; prediction intervals use the Higgins–Thompson–Spiegelhalter method. R 4.5.2, meta 8.2.1.

A. Per-Minute Meta-Analysis, Non-Shockable Rhythm, Survival

**Analysis set**	**Studies**	**k**	**Pooled OR per min (95% CI)**	**I^2 (%)**
All time origins (main-text primary analysis)	Ewy 2015, Hansen 2018, Homma 2019, Enzan 2022	4	0.958 (0.949–0.968)	0.0
Call- or dispatch-anchored only	Ewy 2015, Homma 2019, Enzan 2022	3	0.957 (0.945–0.969)	0.0
Strictly EMS-call-anchored only	Homma 2019, Enzan 2022	2	0.957 (0.945–0.970)	0.0
EMS-arrival-anchored only	Hansen 2018	1	0.960 (0.950–0.980)*	—
Subgroup test, call/dispatch vs EMS arrival	—	4	Q=0.10, df=1, p=0.75	—

*Single study; not pooled. The per-minute estimate for non-shockable rhythm is unchanged by restriction to a common time origin, and the time origin explains none of the (already absent) between-study variation.

B. Categorical Early-Versus-Late Meta-Analysis, CPC 1–2

**Analysis set**	**k**	**Pooled OR (95% CI)**	**95% PI**	**I^2 (%)**
Primary: direct adjusted, all time origins	9	2.76 (2.06–3.71)	0.98–7.79	88.5
restricted to EMS-call-anchored	5	3.07 (2.28–4.13)	1.10–8.54	72.0
restricted to call-	7	2.68 (1.94–3.70)	0.91–7.93	85.6

or CPR-anchored				
excluding the defibrillation-anchored study (Kawakami 2024)	8	2.55 (1.91–3.40)	0.96–6.78	84.6
Comprehensive: all comparisons, all time origins	15	3.19 (2.50–4.06)	1.22–8.31	86.3
restricted to EMS-call-anchored	9	3.78 (2.89–4.95)	1.57–9.13	78.8

Restricting to a common time origin lowered I² from 88.5% to 72.0% in the primary analysis and from 86.3% to 78.8% in the comprehensive analysis, and moved the point estimate by roughly 20% in either direction depending on which origin was retained. Non-equivalent time origins are therefore a substantial, but not the sole, source of the heterogeneity. Every restriction remained statistically significant and in the same direction.

C. Time Origin as a Subgroup Variable

**Analysis**	**Time origin**	**k**	**Pooled OR (95% CI)**	**I² (%)**
Primary categorical (direct adjusted)	Emergency call	5	3.07 (2.28–4.13)	72.0
	CPR initiation	2	1.85 (0.98–3.47)	79.9
	EMS arrival	1	1.87 (1.39–2.52)	—
	First defibrillation	1	4.76 (3.74–6.07)	—
Four-group test		9	Q=25.69, df=3, p<0.001	
Two-group test (call-anchored vs other)		9	Q=0.63, df=1, p=0.43	
Comprehensive, two-group test (call vs other)		15	Q=3.27, df=1, p=0.071	

The four-group test is significant but rests on two single-study subgroups and is therefore fragile; the more robust two-group contrast is not significant. Read together, these tests indicate that the time origin contributes materially to the spread of the categorical estimates without being the only driver of it.

D. Rhythm Contrast Within a Time-Homogeneous Subset

**Rhythm subgroup**	**k**	**Pooled OR (95% CI)**	**I² (%)**
Shockable	3	4.61 (2.86–7.44)	85.8

Non-shockable	4	3.16 (2.22–4.48)	57.9
Overall (not rhythm-stratified)	2	3.75 (1.61–8.75)	89.5
Between-rhythm test	9	Q=1.58, df=2, p=0.46	

Comprehensive categorical analysis restricted to the nine estimates anchored on the emergency call. The shockable-versus-non-shockable difference that is statistically significant in the full comprehensive set (p=0.020) is no longer apparent once the contributing studies share a single time origin (p=0.46). The rhythm contrast therefore depends both on including lower-quality indirect and unadjusted comparisons and on pooling across non-equivalent time origins, and is reported in the main text as hypothesis-generating rather than established.

Note on the restricted cubic spline analyses. A corresponding restriction was not feasible for the time-response models. Each spline rests on four studies per rhythm, of which at most two share a time origin (shockable: Fukuda 2016 and Otani 2025, both call-anchored; non-shockable: Goto 2013 and Funada 2018, both CPR-anchored), which is too few to fit and pool a three-knot restricted cubic spline. The heterogeneity of time origins in those analyses is therefore acknowledged as an unresolved limitation rather than addressed analytically, and the curves are reported as exploratory throughout.

Table S6. E-value Sensitivity Analysis for Unmeasured Confounding

Analysis	Rhythm	Outcome	OR (95% CI)	E-value–point (OR≈RR)	E-value–lower CI	E-value–point (√OR)
**Categorical primary (direct adjusted)**	Pooled	Neuro	2.76 (2.06–3.71)	4.97	3.53	2.71
**Categorical primary (direct adjusted)**	Non-shockable	Neuro	2.45 (2.04–2.94)	4.33	3.50	2.51
**Categorical primary (direct adjusted)**	Shockable	Neuro	3.81 (2.52–5.77)	7.08	4.48	3.32
**Comprehensive (all comparisons)**	Shockable	Neuro	4.31 (3.29–5.65)	8.10	6.04	3.57
**Comprehensive (all comparisons)**	Non-shockable	Neuro	2.80 (2.21–3.56)	5.05	3.84	2.74
**Categorical survival (pooled)**	Pooled	Survival	2.08 (1.48–2.93)	3.58	2.31	2.24
**Indirect (Fukuda 2016)**	Shockable	Neuro	7.50 (NR)	14.48	—	4.92
**Adjusted single (Sagisaka 2017)**	Overall	Neuro	5.92 (NR)	11.32	—	4.30

**Per-minute delay (per 1 min)**	Non-shockable	Survival	0.958 (0.949–0.968)	1.26	1.22	1.17
**Per-minute delay (per 1 min)**	Non-shockable	Neuro	0.953 (0.929–0.978)	1.28	1.17	1.18
**5-min cumulative delay (OR^5)**	Non-shockable	Survival	0.81 (0.77–0.85)	1.78	1.64	1.47
**5-min cumulative delay (OR^5)**	Non-shockable	Neuro	0.79 (0.69–0.89)	1.86	1.48	1.51

E-value = the minimum strength of association, on the risk-ratio scale, that an unmeasured confounder would need with both epinephrine timing and outcome, over and above the measured covariates, to fully explain away the observed association; the lower-CI E-value is the strength needed to move the confidence-interval limit nearest the null to 1.0. The primary E-values assume a rare outcome (OR≈RR, justified by event rates <5%); the final column applies the more conservative square-root transformation (RR≈√OR). Larger E-values are conventionally read as greater robustness to confounding; in this setting, however, confounders attaining RR 3–7 (notably resuscitation-time bias) are plausible, so the largest associations, particularly for shockable rhythm, are interpreted as the quantitative signature of residual confounding rather than as causal effects. Per-minute estimates are on a per-1-minute scale and are not directly comparable to the categorical early-versus-late contrasts; the OR^5 rows show the cumulative effect of a 5-minute delay. NR = not reported (single-study estimates lacked a published 95% CI). Computed with the EValue R package (rare=TRUE and rare=FALSE).

Table S7. Sensitivity Analyses for Categorical CPC 1–2 Meta-Analysis

Analysis	k	Pooled OR (95% CI)	I^2 (%)
**Primary: Direct adjusted comparisons**	**9**	**2.76 (2.06–3.71)**	**88.5**
All comparisons (comprehensive)	15	3.19 (2.50–4.06)	86.3
Direct comparisons only (incl. crude)	11	3.00 (2.30–3.92)	88.7
Adjusted OR only	9	2.74 (1.99–3.77)	88.0
Excluding crude ORs	13	3.03 (2.32–3.96)	85.9
Excluding risk ratios	13	3.30 (2.48–4.38)	87.7
Non-overlapping registries only	8	3.12 (2.20–4.44)	91.9
Excluding Sagisaka 2017 (FDMA overlap)	14	3.05 (2.40–3.89)	87.2
Excluding Tanaka 2016 (FDMA overlap)	14	3.26 (2.51–4.23)	88.1
Non-critical ROBINS-I only	15	3.19 (2.50–4.06)	86.3

All sensitivity analyses maintained statistical significance and consistent effect direction (OR range 2.74–3.30 across the analyses in this table, and 2.55–3.78 once the time-origin restrictions of Table S5 are included). I^2 remained high (86–92%) across all analyses, indicating heterogeneity was not driven by any single methodological factor. The two FDMA-overlap sensitivity analyses (excluding either Sagisaka 2017 or Tanaka 2016, the two overlapping mixed-rhythm registry studies that both contribute to the overall subgroup) bracketed the comprehensive estimate (3.05–3.26), confirming that the result is not an artifact of registry double-counting. The non-critical ROBINS-I sensitivity yielded results identical to the comprehensive analysis because the only critical-risk study (Koscik 2013) was already excluded from quantitative synthesis.

Table S8. Leave-One-Out Sensitivity Analysis (Per-Minute Non-Shockable Survival, k=4)

Study Omitted	Pooled OR (95% CI)	I^2 (%)
Ewy 2015	0.96 (0.95–0.97)	0
Hansen 2018	0.96 (0.94–0.97)	0
Homma 2019	0.96 (0.95–0.97)	0
Enzan 2022	0.96 (0.95–0.97)	0

All leave-one-out estimates remained stable at OR 0.96 with I^2=0%, confirming that no single study disproportionately influenced the pooled result.

Table S9. ROBINS-I Risk of Bias Assessment

Study	D1: Confounding	D2: Selection	D3: Classification	D4: Deviations	D5: Missing Data	D6: Measurement	D7: Reporting	Overall
Hayashi 2012	Serious	Low	Moderate	Low	Moderate	Low	Low	Serious
Nakahara 2012	Serious	Low	Moderate	Low	Low	Low	Low	Serious
Goto 2013	Serious	Low	Moderate	Low	Low	Low	Low	Serious
Koscik 20	Critical	Moderate	Moderate	Low	Moderate	Low	Moderate	Critical

Study	D1: Confounding	D2: Selection	D3: Classification	D4: Deviations	D5: Missing Data	D6: Measurement	D7: Reporting	Overall
13								
Cantrell 2013	Serious	Moderate	Moderate	Low	Moderate	Low	Moderate	Serious
Ewy 2015	Serious	Moderate	Low	Low	Moderate	Low	Low	Serious
Hubble 2015	Serious	Moderate	Moderate	Low	Moderate	Low	Low	Serious
Fukuda 2016	Serious	Low	Moderate	Low	Low	Low	Low	Serious
Tanaka 2016	Serious	Low	Moderate	Low	Low	Low	Low	Serious
Hubble 2017	Serious	Moderate	Moderate	Low	Moderate	Low	Low	Serious
Sagisaka 2017	Serious	Low	Moderate	Low	Low	Low	Low	Serious
Funada 2018	Serious	Low	Moderate	Low	Low	Low	Low	Serious
Hansen 2018	Serious	Low	Low	Low	Low	Low	Low	Serious
Homma	Serious	Moderate	Serious	Low	Moderate	Low	Moderate	Serious

Study	D1: Confounding	D2: Selection	D3: Classification	D4: Deviations	D5: Missing Data	D6: Measurement	D7: Reporting	Overall
2019								
Sigal 2019	Serious	Moderate	Moderate	Low	Moderate	Low	Low	Serious
Perkins 2020	Moderate	Low	Low	Low	Low	Low	Low	Moderate
Okubo 2021	Moderate	Low	Low	Low	Low	Low	Low	Moderate
Enzan 2022	Serious	Moderate	Moderate	Low	Low	Low	Low	Serious
Fukuda 2021	Serious	Low	Moderate	Low	Low	Low	Low	Serious
Kawakami 2024	Serious	Low	Low	Low	Low	Low	Low	Serious
Sakamoto 2025	Moderate	Moderate	Low	Low	Low	Low	Low	Moderate
Otani 2025	Serious	Low	Moderate	Low	Low	Low	Moderate	Serious
Izumida 2025	Serious	Low	Moderate	Low	Low	Low	Serious	Serious
Okad	Serious	Low	Low	Low	Moderate	Moderate	Low	Serious

Study	D1: Confounding	D2: Selection	D3: Classification	D4: Deviations	D5: Missing Data	D6: Measurement	D7: Reporting	Overall
a 2025								
Gruebl 2025	Serious	Moderate	Low	Low	Moderate	Low	Moderate	Serious

D1: bias due to confounding; D2: bias in selection of participants; D3: bias in classification of interventions; D4: bias due to deviations from intended interventions; D5: bias due to missing data; D6: bias in measurement of outcomes; D7: bias in selection of reported results. Overall risk determined by highest-risk domain. Moderate risk: 3 studies (12%); Serious risk: 21 studies (84%); Critical risk: 1 study (4%). Denominator: all 25 included studies. Domain 1 (confounding) was the predominant driver of serious/critical ratings, reflecting the inability of standard regression to address time-dependent confounding and resuscitation time bias.

Table S10. GRADE Summary of Findings

Population: Adult patients with non-traumatic OHCA | **Intervention:** Earlier epinephrine | **Comparison:** Later epinephrine | **Setting:** EMS (prehospital)

Outcome	k	Effect (95% CI)	Risk of Bias	Inconsistency	Indirectness	Imprecision	Pub. Bias	Certainty
Neuro (CPC), per-min, NS	3	OR 0.953 (0.929–0.978)/min	Serious	Not serious	Not serious	Not serious	Not assessable	Very low
Neuro (CPC), per-min, Overall	2	OR 0.910 (0.900–0.919)/min	Serious	Not serious	Not serious	Not serious	NA	Very low
Survival, per-min, NS	4	OR 0.958 (0.949–0.968)/min	Serious	Not serious	Not serious	Not serious	Not assessable	Very low
Survival, per-min, S	2	OR 0.980 (0.904–1.061)/min	Very serious	Very serious	Not serious	Very serious	NA	Very low
Neuro (CPC), categorical, All	15	OR 3.19 (2.50–4.06)	Serious	Serious	Serious	Not serious	NA	Very low

Outcome	k	Effect (95% CI)	Risk of Bias	Inconsistency	Indirectness	Imprecision	Pub. Bias	Certainty
Neuro (CPC), categorical, S	5	OR 4.31 (3.29–5.65)	Serious	Serious	Serious	Not serious	NA	Very low
Neuro (CPC), categorical, NS	6	OR 2.80 (2.21–3.56)	Serious	Not serious	Not serious	Not serious	NA	Very low
Survival, categorical, All	6	OR 2.08 (1.48–2.93)	Serious	Serious	Serious	Not serious	NA	Very low

All evidence from observational studies (starting certainty: Low). Risk of bias assessed using ROBINS-I: serious risk for most outcomes due to residual confounding from time-dependent treatment assignment and resuscitation time bias; very serious for shockable per-minute analysis (k=2, opposing results). Large effect and dose-response gradient considered for upgrading but judged insufficient to offset downgrading factors. NS = non-shockable, S = shockable, NA = not assessable.

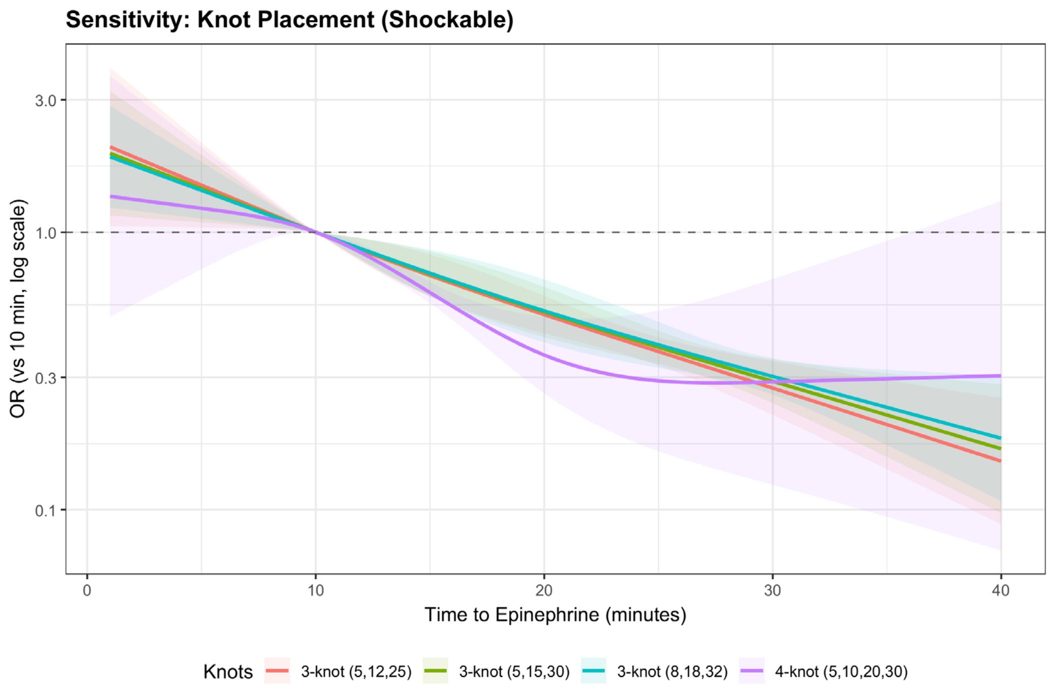

Figure S1. Sensitivity analysis for RCS knot placement.

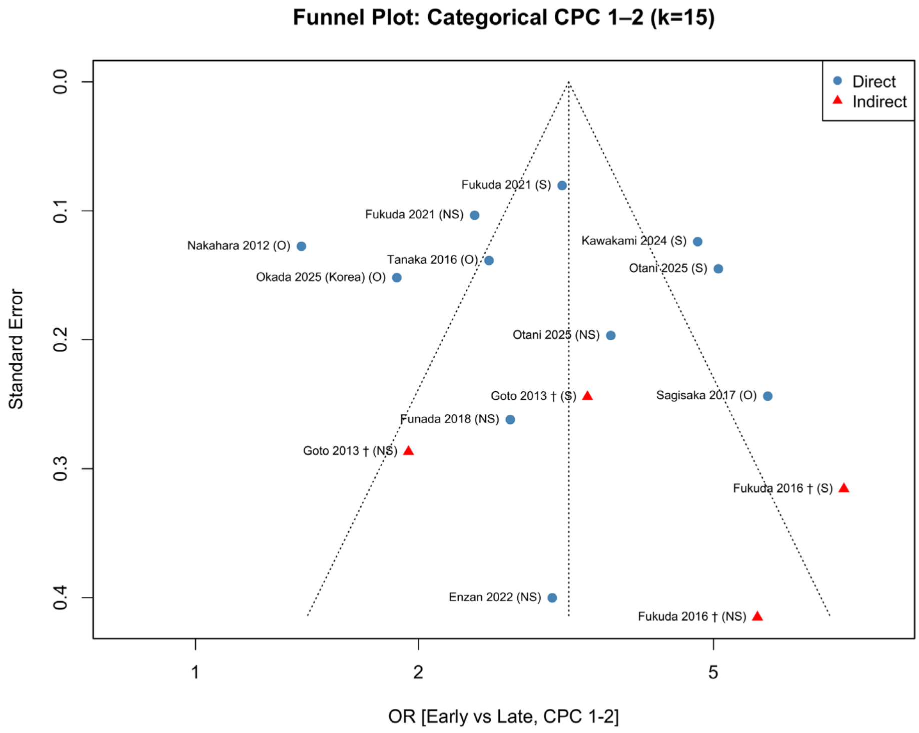

Figure S2. Funnel plot for categorical CPC 1–2 meta-analysis (k=15). Direct comparisons shown as blue circles; indirect comparisons (†) shown as red triangles. Study labels include rhythm abbreviation (S=shockable, NS=non-shockable, O=overall). The four indirect estimates (Goto 2013 and Fukuda 2016) lie outside the pseudo-95% confidence region, consistent with the known upward bias of indirect OR ratios rather than selective publication.

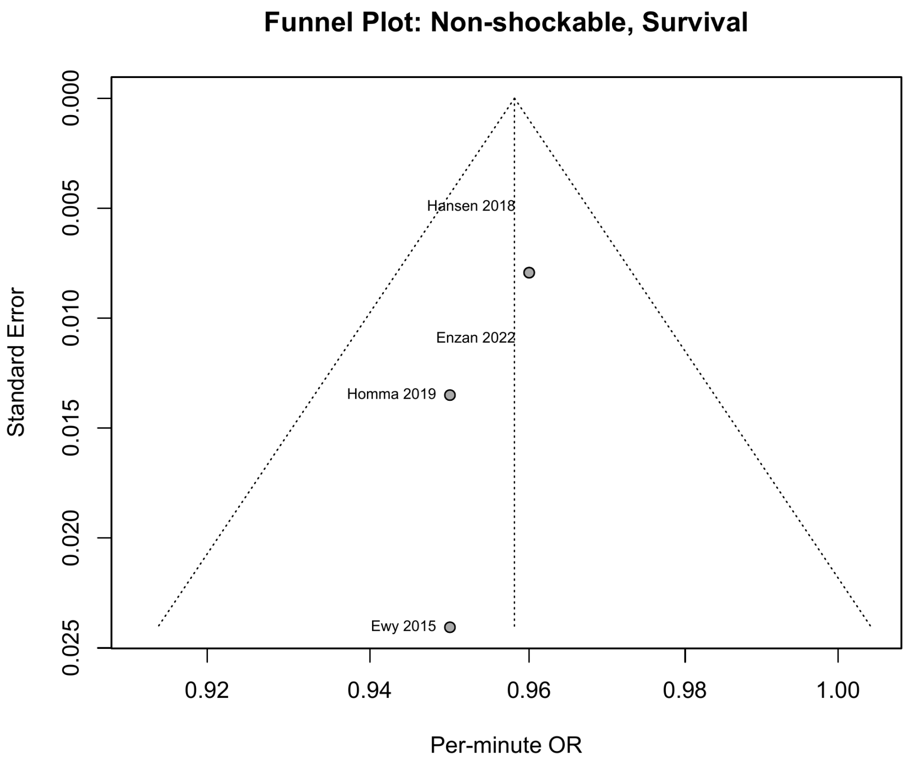

Figure S3. Funnel plot for per-minute non-shockable survival meta-analysis (k=4). With only four studies, this plot is presented for visual inspection only; formal tests of funnel-plot asymmetry are underpowered and unreliable with fewer than 10 studies, so publication bias could not be assessed [47].

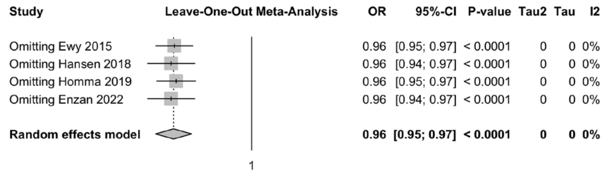

Figure S4. Leave-one-out sensitivity analysis for per-minute non-shockable survival.

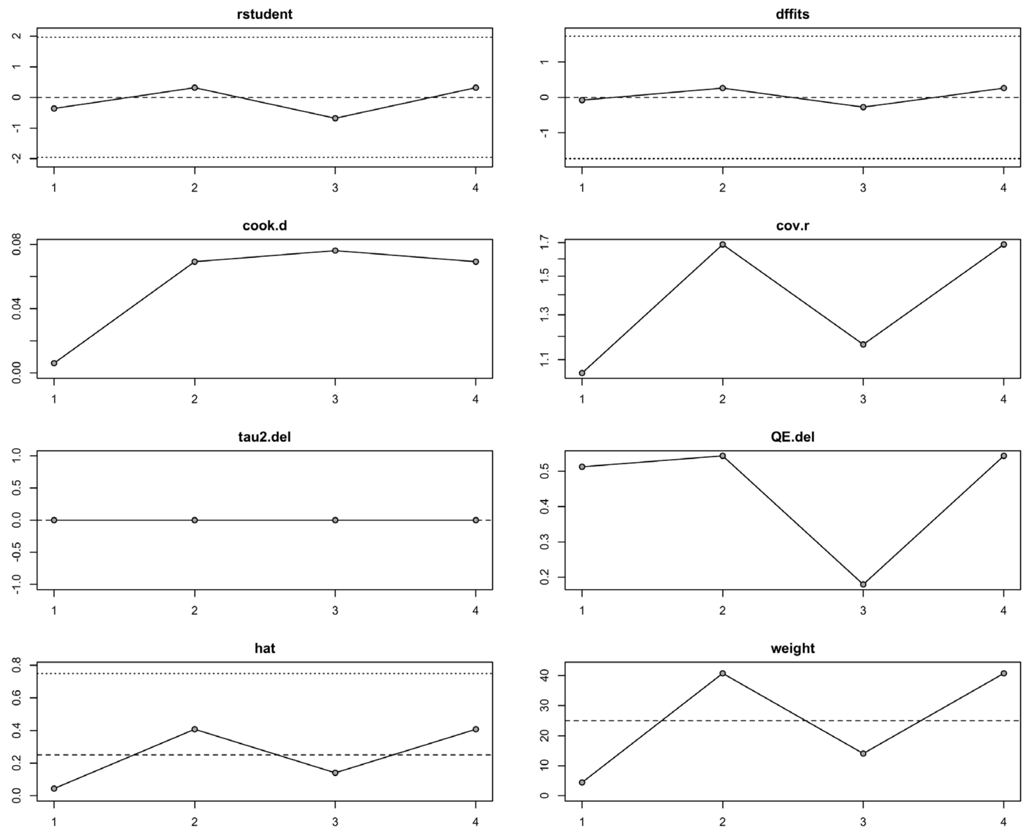

Figure S5. Influence diagnostics for per-minute non-shockable survival.

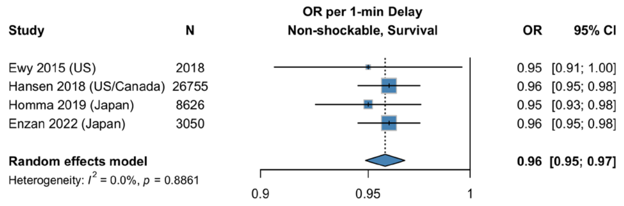

Figure S6. Forest plot: Per-minute OR for non-shockable survival (k=4).

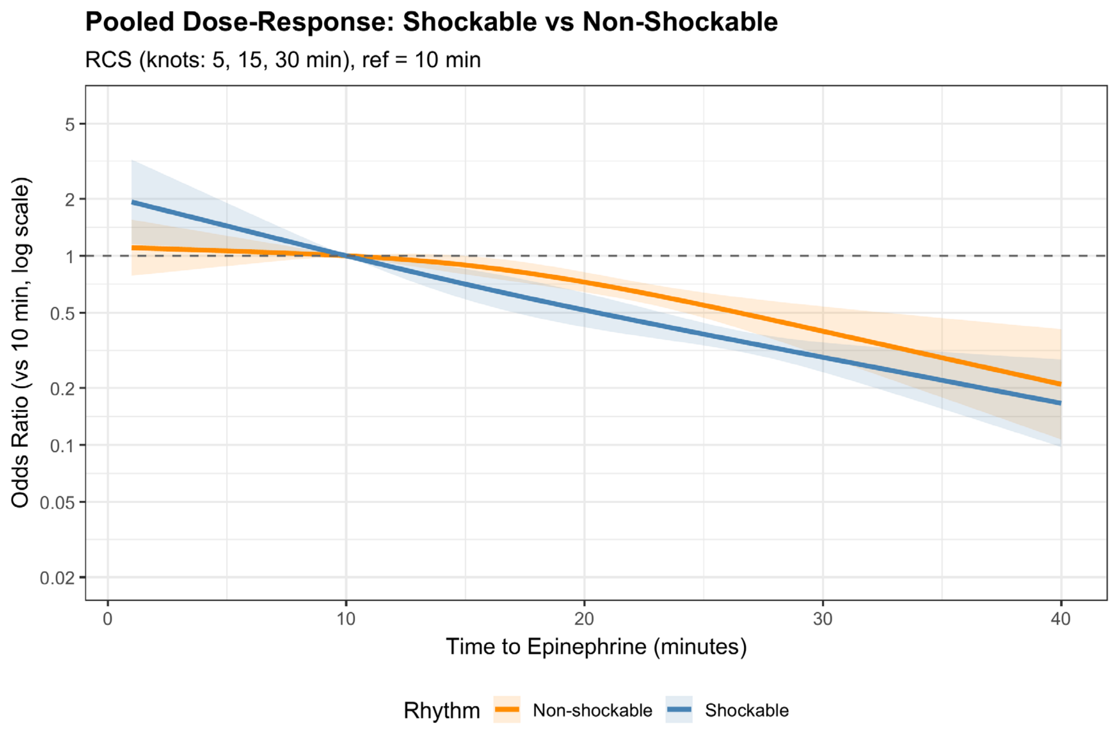

Figure S7. Combined time-response comparison: shockable vs. non-shockable rhythm restricted cubic spline curves overlaid.
